# Supplementary material for: Highly specific σ2R/TMEM97 ligand FEM-1689 alleviates neuropathic pain and inhibits the integrated stress response
Source: Proc Natl Acad Sci U S A. 2023 Dec 20;120(52):e2306090120. doi: 10.1073/pnas.2306090120 (PMC10756276; doi:10.1073/pnas.2306090120)
Supplement: Supplementary file 1 — Appendix 01 (PDF) [file pnas.2306090120.sapp.pdf]

Supplementary Figures and Tables:

A

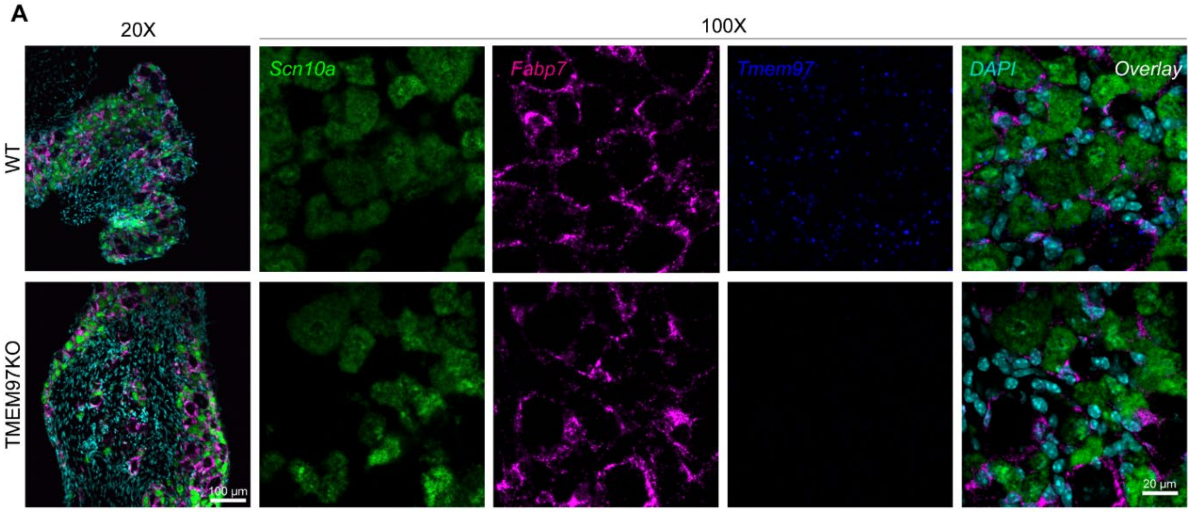

B

Name	Description	Tmem97
PSPEP8	Peptidergic (TrpM8), DRG	0.0357
PSPEP7	Peptidergic (TrpM8), DRG	0.0314
PSPEP6	Peptidergic (TrpM8), DRG	0.0373
PSPEP5	Peptidergic (PEP1.2), DRG	0
PSPEP2	Peptidergic (PEP1.3), DRG	0.069
PSPEP4	Peptidergic (PEP1.1), DRG	0.0773
PSPEP3	Peptidergic (PEP1..4), DRG	0.0875
PSPEP1	Peptidergicv (PEP2), DRG	0.0514
PSNF3	Neurofilament (NF2/3), DRG	0.0323
PSNF2	Neurofilament (NF4/5), DRG	0.053

Name	Description	Tmem97
PSNF1	Neurofilament (NF1), DRG	0.053
PSNP1	Non-peptidergic (TH), DRG	0.057
PSNP2	Non-peptidergic (NP1.1), DRG	0.195
PSNP3	Non-peptidergic (NP1.2), DRG	0.301
PSNP4	Non-peptidergic (NP2.1), DRG	0.156
PSNP5	Non-peptidergic (NP2.2), DRG	0.057
PSNP6	Non-peptidergic (NP3), DRG	0.152
SCHW	Schwann cells	0.299
SATG2	Satellite glia	0.197
SATG1	Satellite glia, proliferating	0.77

C

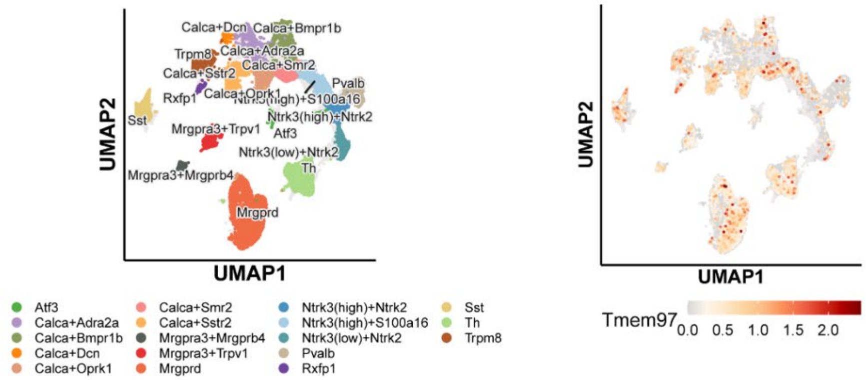

Supp Fig 1. RNAScope *in situ* hybridization of *Tmem97*, *Fabp7*, and *Scn10a* transcripts in mouse DRGs. (A) *Tmem97* is expressed in *Scn10a*-expressing nociceptors and *Fabp7*-expressing satellite glial cells in DRGs obtained from wild-type (WT) animals. *Tmem97* expression is lost in TMEM97-knockout (KO) DRGs. (B) Publicly available single-cell RNA sequencing dataset from mousebrain.org (1) shows that *Tmem97* is expressed across all neuronal subtypes. *Tmem97* expression is enriched in non-peptidergic neurons, Schwann cells, and satellite glial cells. (C) A recently published Harmonized Atlas of the DRG (2) integrated single-cell RNA sequencing data from humans, non-human primates, and rodents. *TMEM97* expression was found across neuronal populations in this dataset.

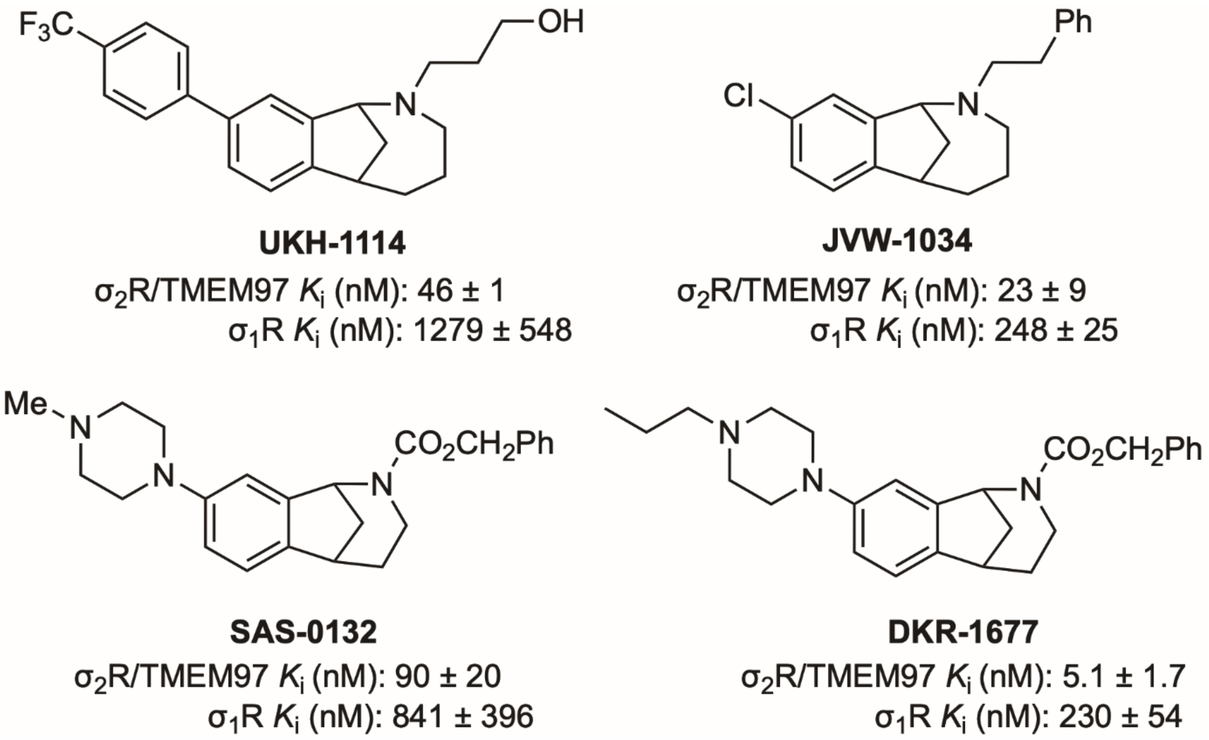

Supp Figure 2. Structures and binding affinities of selected biologically-active methanobenzazocines (e.g. UKH-1114 and JVW-1034) and norbenzomorphans (e.g. SAS-0132 and DKR-1677). K_i values were determined at the PDSP using σ_2R/TMEM97 sourced from rat PC12 cells and σ_1R sourced from guinea pig brain, and values are reported as an average ± standard deviation of two or more independent experiments (3).

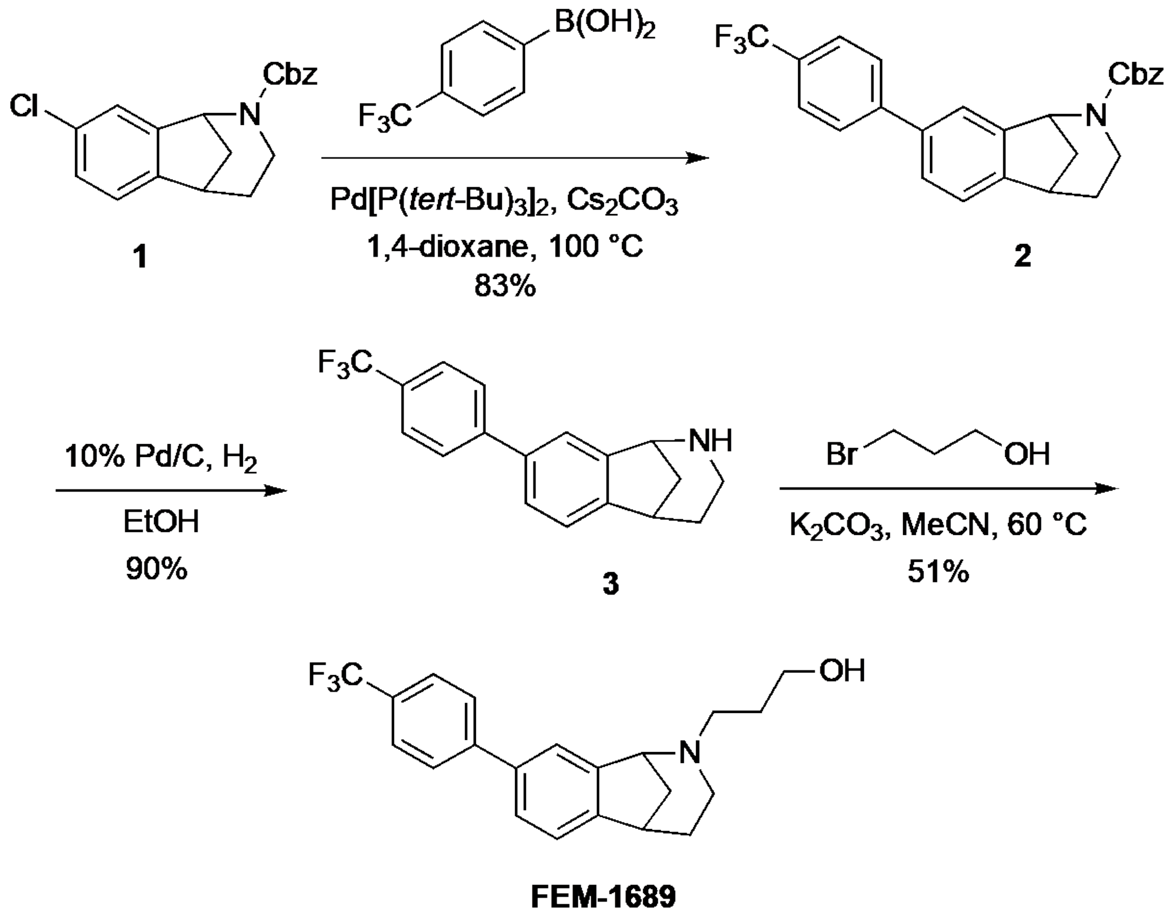

Supp Fig 3. Synthesis of FEM-1689.

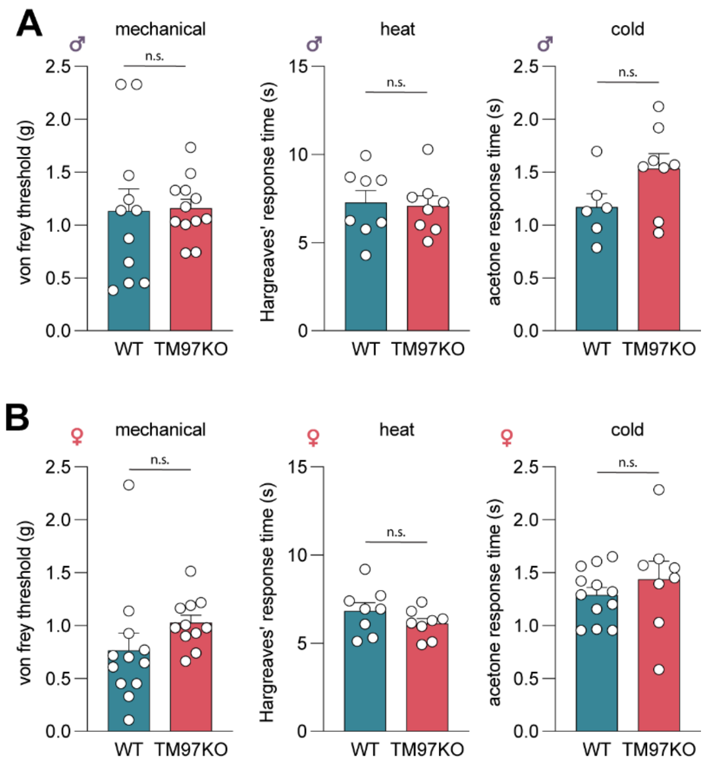

Supp Fig 4. Male (A) and female (B) wild-type (WT) and TMEM97KO (TM97KO) animals have similar mechanical (von Frey test), heat (Hargreaves' test), and cold (acetone test) sensitivity under naïve, baseline conditions. A two-way student's t-test was used to determine statistical significance: n.s. indicates "not significant".

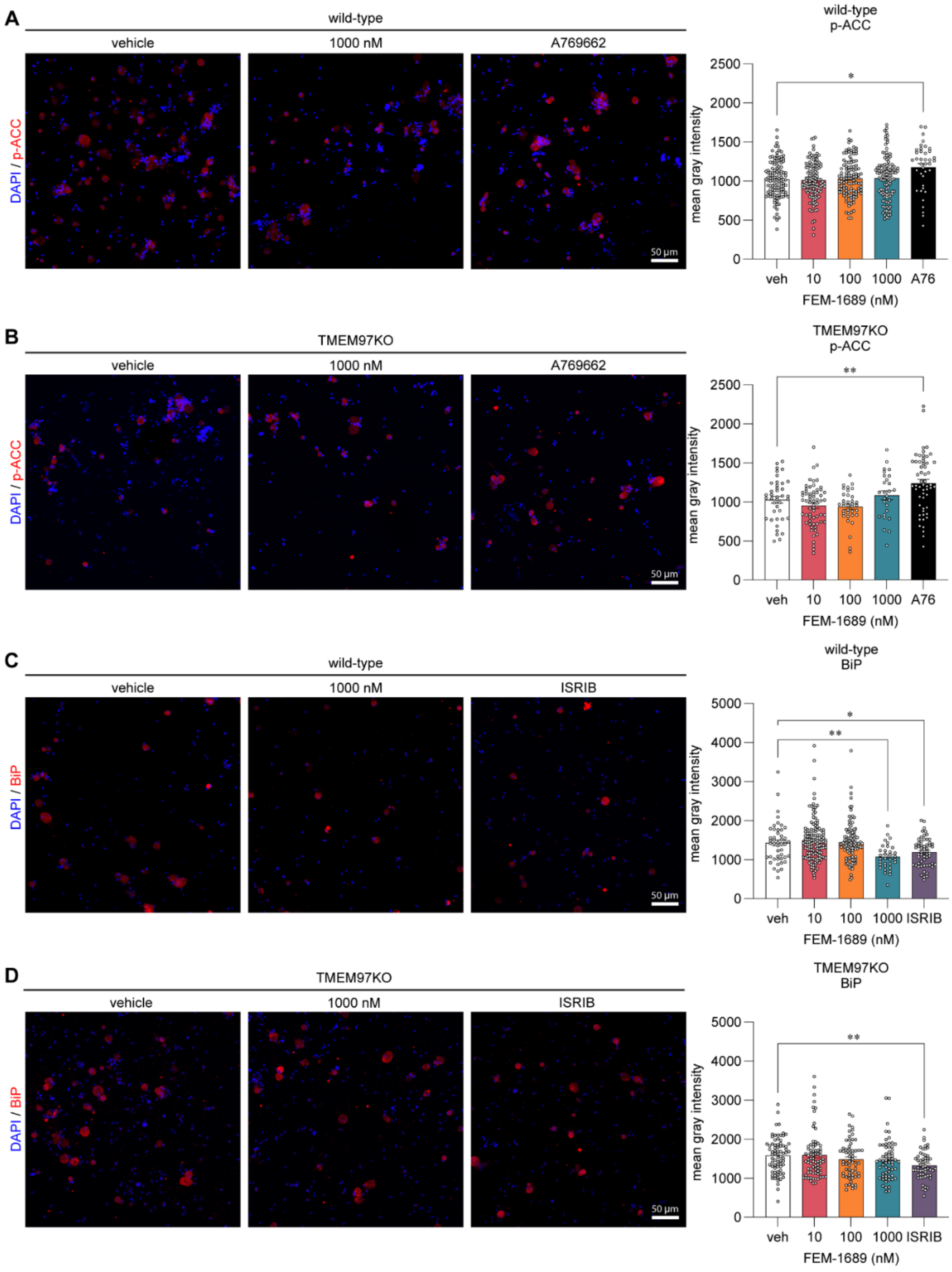

Supp Fig 5. Wild-type (WT) and TMEM97KO DRG neurons were treated with FEM-1689 and probed for changes in p-ACC and BiP levels using immunocytochemistry. (A, B) p-ACC levels remained unchanged in WT and TMEM97KO neurons treated with FEM-1689. A769662 (100 μM), an AMPK agonist, was able to elevate p-ACC levels in both wild-type and TMEM97KO neurons. (C, D) BiP levels in wild-type neurons were only reduced at a high concentration of 1000 nM of FEM-1689. BiP levels in TMEM97KO neurons were unaffected. ISRIB (200 nM) treatment was able to reduce BiP levels in both wild-type and TMEM97KO neurons. One-way ANOVA followed by Tukey's post hoc test $^{*}p<0.05$, $^{**}p<0.01$.

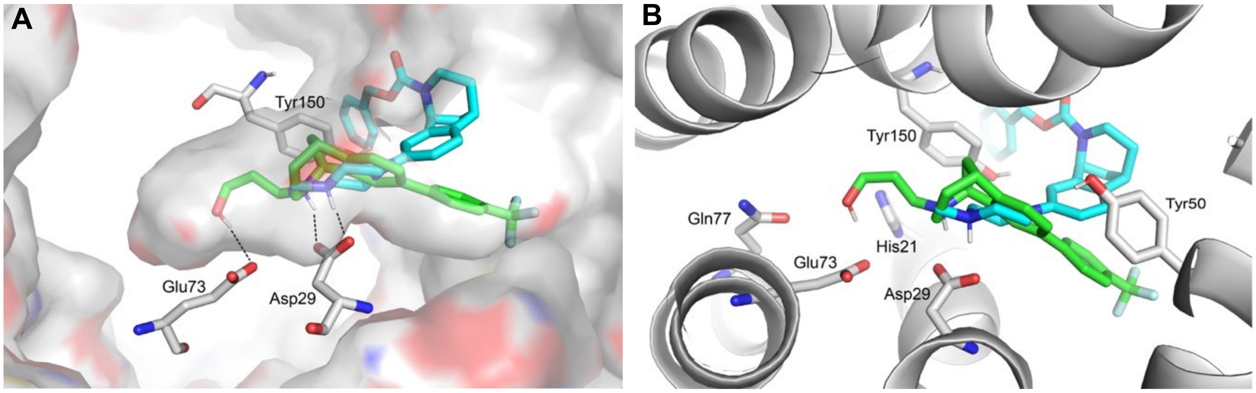

Supp Fig 6. Glide docking of the predicted top ranked poses of 1*S,5R*-enantiomers of norbenzomorphans SAS-0132 and FEM-1689 in the σ_2R/TMEM97 binding site with selected residues shown. The σ_2R/TMEM97 structure used for docking was the 2.71 Å structure of the bovine receptor bound to roluperidone (7M94); Chain C was used for docking studies (4). Nitrogen and oxygen atoms are colored blue and red, respectively, and the protein is represented by grey surface or cartoon and sticks for selected residues. Except for some polar hydrogen atoms on selected residues only polar hydrogen atoms of the ligands are shown. In the binding pocket of σ_2R/TMEM97, each ligand adopts a bound conformation in which the carboxyl group of Asp29 is positioned in close proximity to the protonated nitrogen atom of the ligand. The protonated amine in each ligand is also predicted to engage in a conserved cation-π interaction with Tyr150. Despite the similar electrostatic interactions between the protonated amino groups with Asp29 and Tyr150, other interactions between SAS-0132 and FEM-1689 within the internal binding pocket and with the external surface of σ_2R/TMEM97 are predicted to be significantly different. The bound poses of SAS-0132 and its homolog DKR-1677 are predicted to be identical due to their comparable structure. (A) Overlay of (1*S*,5*R*)-SAS-0132 (cyan) and (1*S*,5*R*)-FEM-1689 (green) docked with σ_2R/TMEM97 showing the protein surface (grey, red, blue), the binding pocket, the protonated nitrogen atoms of the ligands, and the side chains of protein residues Asp29, Glu73 and Tyr150. Interactions between Asp29 with the protonated amino groups of ligands are shown by dashed black lines. A weaker interaction between the hydroxyl group on the side chain of FEM-1689 with Glu73 is also shown by a dashed black line. (B) Overlay of (1*S*,5*R*)-SAS-0132 (cyan) and (1*S*,5*R*)-FEM-1689 (green) docked with σ_2R/TMEM97 showing the protein backbone (grey cartoon), the protonated nitrogen atoms of the ligands, and the side chains of protein residues His21, Asp29, Tyr50, Glu73, Gln77, and Tyr150, which are key residues in the binding pocket.

Supp Table 1. Binding profile of FEM-1689 at non-sigma receptor sites. Competitive binding assays were completed by the Psychoactive Drug Screening Program (PDSP) at the University of North Carolina at Chapel Hill. Percent inhibition of radioligand at targets is determined for FEM-1689. Ki's are determined with secondary assay for any hits with greater than 50% binding in the primary assay. hERG binding is included as a safety/nuisance target.

Target	K_i (nM)	Target	K_i (nM)
**$5HT_{1A}$**	*	Beta3	*
**$5HT_{1B}$**	*	BZP Rat Brain	6595
**$5HT_{1D}$**	*	D_1	*
**$5HT_{1e}$**	*	D_2	*
**$5HT_{2A}$**	*	D_3	*
**$5HT_{2B}$**	*	D_4	*
**$5HT_{2C}$**	*	D_5	*
**$5HT_3$**	*	DOR	*
**$5HT_{5a}$**	*	GabaA	*
**$5HT_6$**	>10,000	H_1	*
**$5HT_7$**	*	H_3	*
**$Alpha_{1a}$**	1994	H4	5512
**$Alpha_{1b}$**	*	KOR	7091 ± 2395[a]
**$Alpha_{1d}$**	*	M_1	*
**$Alpha_{2a}$**	*	M_2	*
**$Alpha_{2b}$**	*	M_3	*
**$Alpha_{2c}$**	*	M_4	*
**Beta1**	*	M_5	*
**Beta2**	*	MOR	*
**PBR**	*	NET	405 ± 227[b]
**SERT**	*	hERG	839

∗ < 50% inhibition of radioligand binding at 10 μM test article.

[a] Average of two K_i determinations; [b] Average of four K_i determinations; all other K_i values are from a single determination

Supp Table 2. Demographics of organ donors and their cause of death.

ID	Sex	Age	Cause of Death	Experiment
AIGD395	F	45	CVA/Stroke	RNAScope (Fig 1 A-E)
AJEK100	M	22	Overdose/CVD	RNAScope (Fig 1 A-E)
AJHX028	F	19	Anoxia/Overdose	RNAScope (Fig 1 A-E)
AJAM174	F	25	Head Trauma/MVA	Cultured DRG neurons (Fig 6 A-B)
AJCI494	F	40	Anoxia/CVA	Cultured DRG neurons (Fig 6 A-D)
AJDS185	F	16	Head Trauma	Cultured DRG neurons (Fig 6 C-D)

*MVA: motor vehicle accident, CVA: cerebrovascular accident, CVD: cardiovascular disease

Supp Table 3. Number of neurons analyzed for *in vitro* mouse DRG studies

Figure	Genotype	Condition	N
Fig 4D	Wild-type	Vehicle	55
		10 nM	22
		30 nM	47
		100 nM	36
		300 nM	24
		1000 nM	21
		ISRIB	66
Fig 4E	TMEM97KO	Vehicle	50
		10 nM	33
		30 nM	65
		100 nM	34
		300 nM	39
		1000 nM	36
		ISRIB	89
Fig 4F-G	Wild-type	0.5h	197
		1h	145
		3h	106
		6h	103
		12h	140
		16h	130
Fig 4H-J	Wild-type	Vehicle	10
		100 nM	8
Fig 4K-M	TMEM97KO	Vehicle	10
		1000 nM	12
Fig 5A-C	Wild-type	Vehicle	82
		10 nM	61
		30 nM	63
		100 nM	86
		300 nM	54
Fig 5D-F	Wild-type	Vehicle	83
		10 nM	101
		30 nM	102
		100 nM	57
		300 nM	80

Supp Table 4. Number of neurons analyzed for in vitro human DRG studies

Figure	Condition	N
Fig 8A, B	Vehicle	65
	10 nM	103
	100 nM	133
Fig 8C, D	Vehicle	71
	Vehicle + MGO	103
	100 nM + MGO	95

Supplementary Methods:

Animals

TMEM97KO mice were donated by Dr. Liebl (University of Miami, Mouse Resource & Research Centers MMRRC, $Tmem97^{tm1(KOMP)Vlcg}$, stock #050147-UCD) (5). A colony of these animals were maintained at University of Texas at Dallas. Knockout and wild-type animals were obtained from the same colony. Genotyping was performed using PCR (WT: Fwd: CCAATCCTCTACACACTCCTGTT Rev: CTGGTGGCCGTCCCTATTT, Mutant: Fwd: ACTTGCTTTAAAAAACCTCCCACA. Rev: TCCTTCCCTGTAACCCATTTCTGGC). Animals were maintained on a 12-hour light-dark cycles. Adult male and female mice at least 12-week-old were used throughout the experiment. All experiments were in accordance with the National Institutes of Health guidelines and the Animal Care and Use Committee at the University of Texas at Dallas (protocol # 14-04).

Spared nerve injury model of neuropathic pain

Spared nerve injury (SNI) was performed by exposing the sciatic nerve and transecting the peroneal and tibial branches of the nerve, leaving the sural branch intact. Cut nerves were ligated using 4-0 sutures (Kent Scientific #SUT-15-2) and the wound was closed using surgical staples. In sham animals, an incision was made to expose the nerves, but the nerves were not transected. SNI and sham surgeries were completed on the same day in a randomized order. SNI and sham data were obtained from two separate cohorts of nerve injury experiments. Animals were anesthetized with isoflurane/oxygen (50:50) mixture delivered through a nose cone. We ensured that pain reflexes were lost prior to the surgery. A 100 µL subcutaneous injection of 10% gentamicin (Sigma-Aldrich #G1272) was used to prevent infections. Animals were monitored daily and tested for mechanical sensitivity at days 7, 10, and 14 post-surgery. Behavioral testing was technically completed blinded to surgical treatment (SNI vs sham) although SNI animals often exhibit some cupping of the paw making a full blinding impossible.

Mouse pain behavior assay – Mechanical, cold and heat sensitivity

Baseline testing was completed four days prior to SNI/sham surgery (**Fig 4**). Animals were habituated for at least an hour in their apparatus before being tested. Experimenters were blinded to genotype for all behavior assays. Following SNI, blinding to the injury was not possible because animals will SNI develop a “cupped” hind paw. Blinding to genotype was still maintained. Mechanical paw withdrawal thresholds in mice were assessed using the Simplified Up-Down (SUDO) method of von Frey filaments test (6). von Frey filaments were obtained from Ugo Basile and were calibrated in the lab using a weigh scale (VWR, 314AC) to 0.01 grams. Lateral surface of the paws was tested at baseline and following SNI.

Cold sensitivity was assessed using drops of acetone applied to the plantar surface of each paw. Pain-like behaviors (i.e. vigorous shaking and licking) were timed for up to 45 seconds. Each paw was tested three times and both paws were averaged.

Heat latency to respond was measured using a Hargreaves device (7) (IITC Model 400, Life Science Inc.; Harvard Apparatus, CA) with the heated glass set at 29 °C, 40% active laser power, and a 20-second cutoff. Each paw was tested three times and both paws were averaged. Light was targeted to the lateral surface of the paws.

Human samples – DRG culturing

In collaboration with the Southwest Transplant Alliance, human dorsal root ganglia (DRG) were obtained from organ donors (**Supp Table 2**). Once extracted, DRGs were either frozen in pulverized dry-ice for RNAScope experiments or immersed in ice-cold N-methyl-D-glutamate-supplemented artificial cerebrospinal fluid (as per (8)) until enzymatic dissociation. Human DRGs were cleaned of any fatty tissue and cut into small 1-mm chunks. These chunks were immersed in 5 ml of pre-warmed enzyme solution (2 mg/mL STEMzyme I, 4 µg/mL DNAse I obtained from Worthington Biochemical #LS004107 and #LS002139) in Hanks’ Balanced Salt Solution (HBSS) without calcium and magnesium (Gibco #14170161). Enzyme solution containing the tissue was immersed in a shaking water bath at 37 °C for 20 min followed by trituration with glass pipettes. This step was repeated 2 - 3 times until the tissue was homogenized. Afterwards, cells were passed through a 100 µm cell strainer and the flow-through containing the cells was

gently layered onto 3 mL of 10% bovine serum albumin (BSA, BioPharm #71-040-002) in HBSS in a 15 ml falcon tube. This BSA gradient containing the cells was centrifuged at 900g for 5 min at room temperature. The pellet containing the cells was resuspended in BrainPhys media (STEMCell #05790) containing 2% SM1 (STEMCell #05711), 1% N2-A (STEMCell #07152), 1% Pen-Strep (ThermoFisher #15070063), and 1% GlutaMax (Gibco #35050061). Human DRG neurons were plated and grown on glass coverslips coated with poly-D-lysine (Sigma #P7405). Cultured DRG neurons were plated for 24 hours before being treated. Methylglyoxal (Sigma-Aldrich #M0252) and FEM-1689 were serially diluted in media.

Mouse samples - DRG cultures

Mouse DRGs were harvested from naïve wild-type and TMEM97KO mice euthanized following isoflurane anesthesia and decapitated. Mouse DRGs were processed and plated as outlined in the human DRG section above with minor differences. Lumbar DRGs of two male and two female mice were cultured together and plated across 4 well replicates of each condition. Mouse DRG neurons were grown in DMEM/F12+GlutaMax (Gibco #10565-018) plus 1% Pen-Strep and 1% N2-A. Cultured cells were plated on glass coverslips for 24 hours before being treated with FEM-1689. SAS-0132 and DKR-1677 were dissolved in 100% DMSO to a final concentration of 200mM and stored at -20 °C. FEM-1689, SAS-0132, and DKR-1677 were serially diluted in media. Untreated media was completely replaced with media containing each compound.

Mouse samples - Neurite outgrowth and Sholl analysis

Cultured mouse DRG neurons from wild-type and TMEM97KO animals were treated with either vehicle or FEM-1689 (100 nM). These cells were immunolabeled with β3 tubulin and DAPI according to the ICC protocol detailed above. Cells were imaged at 20X magnification on a FV3000 confocal microscope (Olympus) and Z-stacked in 1 µm intervals. Images were compiled as maximum Z-stack on ImageJ and transformed to 8-bit images. Sholl analysis was performed using the Neuroanatomy plugin in ImageJ according to the authors recommendations (9). Only solitary cells were used for analysis. The output for each cell was the number of intersections in 1-micron increments. The number of intersections was averaged for each condition across all cells. The area under the curve was calculated using GraphPad Prism.

Mouse samples - RNAscope in situ hybridization

A dorsal root ganglion (DRG) tissue (bilateral L1-L5) was collected from 10-week-old male C57BL6/J wild-type (WT) and littermate-matched TMEM97KO mice. The tissue was embedded in optimal cutting temperature (OCT, TissueTek) and was immediately flash-frozen in dry ice. The DRG were sectioned at 20 microns on a cryostat, and directly mounted onto Super Frost Plus charged slides. Slides were dried at –20 °C for 2 hours to increase tissue adherence and stored at –80 °C until used for RNAscope in situ hybridization.

RNAscope fluorescent in situ hybridization multiplex assay v2 was used as instructed by Advanced Cell Diagnostics (ACDBio Inc). Probes for *Tmem97* (ACD #527591), *Scn10a* [a marker for Nav1.8-expressing nociceptors] (ACD #426011), and *Fabp7* [a marker for satellite glial cells] (ACD #414651) were used to validate the deletion of *Tmem97* expression in TMEM97KO DRGs, and localization of *Tmem97* expression in WT DRGs. Every cohort of slides included at least one slide for negative control with no target probe (ACD #320871) and one positive control slide with three positive control target probe cocktails (ACD #320861) (62) for tissue quality check. Slides were removed from –80 °C and immediately immersed in pre-chilled (4 °C) 10% neutral buffer formalin for 15 min. The slides were rinsed twice in 1X phosphate-buffered saline (PBS, pH 7.4) and dehydrated in a series of different ethanol concentrations of 50%, 70%, and 100% (twice) for 5 min each at room temperature. The hydrophobic barrier was drawn around the section using a hydrophobic pen (ImmEdge PAP pen, Vector Labs) after briefly air drying. Slides were incubated with 1:2 diluted hydrogen peroxide in distilled water for 10 min at room temperature and washed twice in distilled water. The protease IV was applied to each section and incubated for 5 min at room temperature. Slides were washed twice in 1X PBS and RNA scope was performed immediately. A mixture of probes - *Tmem97, Scn10a, and Fabp7* – was hybridized for 2 hours at 40 °C in a humidity control tray

inside a HybEZ oven. Signals for all three channels were amplified using a series of AMPs, and a TSA-based fluorescent label was developed for each channel using Opal 520, 570, and 690 dyes (Akoya Bioscience) for each channel. Slides were cover-slipped with Vectashield anti-fade mounting medium with DAPI. Images were captured using an Olympus FV 3000 confocal microscope at 100X magnification and each image used the same acquisition parameters for WT, TMEM97KO, and negative control slides. Imaging was not completed blinded to genotype.

HEK cells

Human embryonic kidney (HEK) 293T cells (ATCC # CRL-3216) were generously donated by the Campbell Lab (UT Dallas). Cell stocks were previously frozen at 1 million cells/ml in 90% fetal bovine serum (FBS, ThermoFisher #SH300880340) and 10% DMSO. Cells were grown in complete medium (10% FBS, 1% Pen-Strep in DMEM/F12 + GlutaMax (Gibco #10565-018) in T-75 flasks (Greiner Bio-One #658175). Cells were maintained at 37 °C with 5% CO_2 and passaged twice before being plated onto either 96-well or 6-well plates for ICC/spectrophotometry and western blotting, respectively. Cells were counted using the TC20 automated cell counter (Bio-Rad #1450102) using a 1:1 dilution of trypan blue (Gibco #15250061).

In a 96-well plate (ThermoFisher #165305), cells were plated at a density of 20,000 cells per well and grown to roughly 65% confluency before being treated with FEM-1689. For 2 hour FEM-1689 treatment, HEK cells were plated at a density of 32,000 cells per well and treated at roughly 85% confluency. Three to five replicates were made per condition/dose (1/2 log steps ranging from 0.1 nM to 1000 nM). HEK cells were fixed with buffered 10% formalin for 10 min and washed three times with 1X PBS. ICC was performed as outlined previously. Cells were stained for p-eIF2α and DAPI. Immunofluorescence of HEK cells was quantified using the Synergy HTX Multimode Reader. Two filters were used: excitation 360/40nm emission 460/40 (DAPI), and excitation 485/20nm emission 528/20 (p-eIF2α-AlexaFluor 488). Wells were topped with 100 µL of 1X PBS prior to being read. DAPI and p-eIF2α reads were performed at bottom with a gain of 35 and 45, respectively. Area scans of 5x5 matrix was used with 497x497 micron point spacing. Negative control wells did not receive any primary antibody, but the remainder of the protocol was the same. Relative fluorescence units (RFUs) of the negative controls were subtracted from each well. P-eIF2α fluorescence was normalized to DAPI in order to control for the number of cells present in the well and then to the fluorescence of the vehicle treatment as a percentage. Values were plotted in a X-Y plot in GraphPad Prism. Concentrations were transformed to log2 values and data was fit to a non-linear regression curve (inhibitor vs response, variable slope, four parameters) constrained at top at 100, bottom at p-eIF2α levels at 300nM, and IC_{50}>0.

Western blotting

HEK cells were plated at 60,000 cells per well onto six-well plates (ThermoFisher # 351146) for 24 hours before being treated with FEM-1689 overnight. Protein extractions and western blotting was performed as previously published (10). In brief, media from the cells was removed and cells were washed once with 1X PBS. Cells were lysed in the well with radioimmunoprecipitation assay (RIPA) buffer consisting of 25 mM Tris, 150 mM NaCl, 0.1% SDS, 0.5% Na deoxycholate, 1% Triton X-100 with protease and phosphatase inhibitor cocktails (Sigma Aldrich P8340, P5726, P0044 – 1:100 each). Cells were centrifuged at 4 °C at 14,000 rpm for 10 min. The supernatant was used for downstream analysis. Protein was quantified using the Pierce BCA Protein Assay kit (Thermo Scientific, #23225) according to the manufacturer's instructions.

Protein samples were denatured using 4X Laemmli Sample buffer (BioRad #1610747) and heated at 95 °C for 5 min. 20 µg of protein samples were loaded onto 4-20% Stain-Free Criterion TGX gels (BioRad #5678094). Gels were run in Tris/Glycine/SDS running buffer (BioRad #1610772) at 120V for roughly 1.5 hours. Stain-Free gels were activated for 5 min prior to the transfer step. Protein was transferred for 10 min onto low fluorescence polyvinylidene difluoride (PVDF) using a TransBlot Transfer kit (BioRad #1704275) and a Transblot Turbo transfer system (BioRad). Once transferred, total protein was imaged immediately using a ChemiDoc MP system (BioRad). Blots were allowed to dry for 5 min and were blocked with 5% non-fat milk in Tris buffered saline (TBS)-Tween 20 (0.05%). Antibodies were diluted in 1% non-fat milk in TBS-Tween 20. Primary antibodies used in this study were: p-eIF2α (1:1000, Cell Signaling #3398), t-eIF2α (1:1000, Cell Signaling #9722), eIF2A (1:2500, Abcam #ab169528), p-PERK (1:500, Cell Signaling #3192), t-PERK (1:1000, Cell Signaling #3179), BiP (1:1000, Cell Signaling #3177). Goat anti-Rabbit IgG

horseradish peroxidase (HRP) (H+L) (1:10,000) was used as a secondary antibody. Stained blots were imaged using enhanced chemiluminescence (ECL) on a ChemiDoc MP system.

Synthetic procedures and characterization

Binding assays. Sigma receptor binding assays for FEM-1686, which was determined to be >95% pure (LC-MS), were performed by the National Institutes of Mental Health Psychoactive Drug Screening Program (NIMH PDSP) at Chapel Hill, North Carolina (3). σ_1R and σ_2R/TMEM97 were sourced from HEK293T cells transfected with human σ_1R and σ_2R/TMEM97. σ_1R binding affinity (K_i) was determined through competition binding assays with [^{3}H]-(+)-pentazocine, whereas σ_2R/TMEM97 binding affinity (K_i) was determined through competition binding assays using the radioligand [^{3}H]-ditolylguanidine in the presence of (+)-pentazocine to block σ_1R binding sites. K_i values are calculated from an average of two or more independent experiments. Detailed experimental protocols are available on the NIMH PDSP website at https://pdspdb.unc.edu/pdspWeb.

General. Commercial reagents and solvents were used without purification unless stated, but acetonitrile (MeCN) was dried by filtration through two columns of activated molecular sieves. Glassware was dried overnight in an oven at 120 °C or flame dried under vacuum for a minimum of 5 min. All air- or moisture-sensitive reactions were performed under an atmosphere of argon or nitrogen. Reaction temperatures refer to the temperature of the heating or cooling bath. Volatile solvents were removed under reduced pressure using a Büchi rotary evaporator at 25–30 °C. Air- or moisture-sensitive reagents and all solvents were transferred using plastic syringes and steel needles using standard techniques. Proton nuclear magnetic resonance (^{1}H NMR) and carbon nuclear magnetic resonance (^{13}C NMR) spectra were recorded at the indicated field strength in $CDCl_3$. Chemical shifts are reported in parts per million (δ) and are referenced to the deuterated solvent. Coupling constants (*J*) are reported in Hertz (11), and the splitting abbreviations used are: s, singlet; d, doublet; t, triplet; q, quartet; dt, doublet of triplets; ddd, doublet of doublets of doublets; m, multiplet; br s, broad singlet;. Capillary melting points are uncorrected. Accurate mass measurements were determined using an LC-MS system comprised of an Agilent 1260 series HPLC and an Agilent 6530 single quadrupole time-of-flight mass spectrometer. Purities of all compounds submitted for testing at PDSP were determined by LC-MS from the areas under the curves (AUC) at 214 and 254 nm. Column chromatography was performed using glass columns and "medium pressure" silica gel (Silicycle, 230-400 mesh).

*Benzyl 8-(4-(trifluoromethyl)phenyl)-1,3,4,5-tetrahydro-2H-1,5-methanobenzo[c]azepine-2-carboxylate (**2**).* A solution of aryl chloride **1** (12) (327 mg, 1.0 2.0 mmol), 4-trifluoromethylphenylboronic acid (379 mg, 2.0 mmol), Cs_2CO_3 (650 mg, 2.0 mmol), palladium(bis)(*t*-butyl)$_3$ phosphine (25.5 mg, 0.05 mmol) in degassed 1,4-dioxane (4 mL) was stirred for 21 h at 100 °C. The reaction was cooled to room temperature and poured into water (5 mL). The mixture was extracted with CH_2Cl_2 (3 × 15 mL), and the combined organic layers were dried ($MgSO_4$) and concentrated under reduced pressure. The crude product was purified via flash column chromatography (SiO_2), eluting with hexane/EtOAc (50:1 to 20:1 to 13:1 v/v) to afford 367.2 mg (83%) of **2** as a pale yellow oil. ^{1}H NMR (500 MHz, as a mixture of rotamers) δ 7.72-7.24 (comp, 12 H), 5.61 (br s, 0.5 H), 5.49 (br s, 0.5 H), 5.27-5.07 (comp, 2 H), 3.96-3.81 (m, 1 H), 3.39-3.24 (m, 1 H), 2.61-2.43 (m, 1 H), 2.34-2.20 (m, 1 H), 2.12-2.00 (m, 1 H), 1.94 (d, *J* = 11.0 Hz, 1 H), 1.74-1.59 (m, 1 H). ^{13}C NMR (126 MHz, as a mixture of rotamers) δ 155.0, 154.8, 146.5, 144.6, 142.1, 141.9, 139.0, 136.9, 136.8, 129.2 (q, J_{C-F} = 31.5 Hz), 128.4, 127.9, 127.8, 127.3, 125.6 (q, J_{C-F} = 3.8 Hz), 123.3 (q, J_{C-F} = 272.2 Hz), 123.2, 122.7, 122.5, 67.0, 57.6, 57.3, 43.6, 39.5, 38.6, 30.2. HRMS (ESI) *m/z* calcd for $C_{26}H_{22}F_3NNaO_2$ $(M+Na)^+$, 460.1495; found 460.1499.

8-*(4-(Trifluoromethyl)phenyl)-2,3,4,5-tetrahydro-1H-1,5-methanobenzo[c]azepine (**3**).* A mixture of **2** (243 mg, 0.55 mmol), 10% Pd/C (85 mg) and EtOH (4 mL) was stirred under a H_2 balloon at room temperature for 5 h. The mixture was filtered through a pad of Celite, which was washed with CH_2Cl_2 (2 mL), and the combined filtrate and washings were concentrated under reduced pressure. The crude product was purified via flash column chromatography (SiO_2), eluting with MeOH/NEt_3/EtOAc (1:1:8), to afford 150 mg (90%) of **3** as a colorless oil. ^{1}H NMR (500 MHz) δ 7.76 (s, 1 H), 7.68 (q, *J* = 8.5 Hz, 4 H), 7.56 (dd, *J* = 7.5, 1.5 Hz, 1 H), 7.34 (d, *J* = 8.0 Hz, 1 H), 7.11 (br s, 1 H), 4.67 (d, *J* = 2.5 Hz, 1 H), 3.38 (s, 1 H), 3.07 (dd, *J* = 13.0, 5.5 Hz, 1 H), 2.49 (td, *J* = 12.5, 5.0 Hz, 1 H), 2.42 – 2.33 (comp, 2 H), 2.22 (td, *J* = 12.5, 5.0 Hz, 1 H), 1.67 (d, *J* = 13.5 Hz, 1 H). ^{13}C NMR (126 MHz) δ 146.1, 144.2, 139.6, 139.1, 129.3 (q, J_{C-F} = 32.5 Hz), 128.6,

127.4, 125.7 (q, J_{C-F} = 3.8 Hz), 124.2 (q, J_{C-F} = 272.2 Hz), 123.2, 123.1, 58.0, 42.7, 38.8, 38.1, 28.3. HRMS (ESI) *m/z* calcd for $C_{18}H_{17}F_3N$ $(M+H)^+$, 304.1308; found 304.1309.

3-((1S,5R)-8-(4-(Trifluoromethyl)phenyl)-1,3,4,5-tetrahydro-2H-1,5-methanobenzo[c]azepin-2-yl)propan-1-ol (FEM-1689). To a solution of **3** (191.2 mg, 0.63 mmol) in acetonitrile (6 mL) was added K_2CO_3 (261.2 mg, 1.89 mmol), followed by 3-bromopropan-1-ol (175.2 mg, 1.26 mmol). The reaction mixture was heated to 60 °C for 21 h. The reaction mixture was cooled to room temperature and filtered, and the filtrate was concentrated under reduced pressure. The residue was suspended in 1 M aqueous HCl (5 mL) and washed with ether (5 mL). The aqueous phase was basified (pH ~ 8) with 2 M aqueous NaOH and extracted with CH_2Cl_2 (3 × 20 mL). The extracts were combined, dried (Na_2SO_4), filtered, and concentrated reduced pressure. The residue was purified by column chromatography (SiO_2) using CH_2Cl_2:MeOH (30:1 to 15:1) as eluant to yield 115.8 mg (51%) FEM-1689 as a colorless oil. LCMS, retention time 5.31 min, 97% pure.^{1}H NMR (500 MHz) δ 7.69 (s, 4 H), 7.49 (dd, *J* = 7.6, 1.7 Hz, 1 H), 7.39 (s, 1 H), 7.31 (d, *J* = 7.6 Hz, 1 H), 4.16 (d, *J* = 4.6 Hz, 1 H), 3.93 – 3.79 (comp, 2 H), 3.21 (br. s, 1 H), 2.84 (dd, *J* = 11.7, 5.7 Hz, 1 H), 2.77 (ddd, *J* = 12.0, 7.5, 3.8 Hz, 1 H), 2.39 (ddd, *J* = 12.2, 7.8, 3.8 Hz, 1 H), 2.32 – 2.26 (m, 1 H), 2.07 – 1.98 (m, 1 H), 1.97 (d, *J* = 11.0 Hz, 1 H), 1.86 – 1.76 (m, 1 H), 1.75 – 1.66 (m, 1 H), 1.61 – 1.55 (m, 1 H), 1.50 (td, *J* = 11.9, 4.8 Hz, 1 H). ^{13}C NMR (126 MHz) δ 146.9, 145.1, 139.5, 138.4, 129.30 (q, J_{C-F} = 32.4 Hz), 127.5, 127.4, 125.8 (q, J_{C-F} = 3.8 Hz), 124.6 (q, J_{C-F} = 272.4 Hz), 123.2, 123.1, 64.9, 63.6, 56.7, 47.1, 44.7, 39.7, 30.1, 27.2. HRMS (FIA) *m/z* calcd for $C_{21}H_{23}F_3NO$ $(M+H)^+$ 362.1726; found 362.1728.

Glide Docking Calculations. Molecular docking into σ_2R/TMEM97 was performed using the Glide module with standard precision (SP) in Maestro of the Schrodinger software suite (release 2023-1) (13). We have shown that the 1*S,*5*R*-enantiomers of several piperazine-substituted norbenzomorphans have higher affinities for σ_2R/TMEM97 than their 1*R,*5*S*-enantiomers (14), and the known structures of σ_2R/TMEM97 complexed with amine ligands uniformly show the amino groups are protonated (4). Accordingly, LigPrep was used to generate starting ligand structures of the 1*S,*5*R*-enantiomers of SAS-0132 and FEM-1689, and the structures in which the more basic amino group of the ligand is protonated were used. The published structure (2.71 Å; pdb accession 7M94) of bovine σ_2R/TMEM97 complexed with roluperidone has four protomers in the asymmetric unit (4), but Chain C was used for docking studies. All lipids, ions, and waters were removed prior to grid preparation, leaving only the protein and ligand. Hydrogen atoms were added, and the protein was further refined by assigning H-bonds and minimizing energy for the OPLS4 force field. After protein preparation, Asp29 and Glu73 were deprotonated, whereas Tyr150 was protonated. The grid used for docking was centered on the location of the co-crystallized ligand roluperidone, and was 20 Å in the x, y, and z dimensions. During docking computations, a constraint requiring an interaction between a protonated amino group on the ligand and Asp29 was applied because such H-bonds and salt-bridges are conserved in known structures of complexes of σ_2R/TMEM97 with basic amines. Poses were ranked by docking score.

Other Supp Materials:

Dose Response Curves for Receptor Binding for FEM-1689 (PDSP #52656)

Sigma 1 Receptor

PDSP #	LogKi	Ki (nM)
52656	-6.69	205.64
Haloperidol	-8.25	5.69

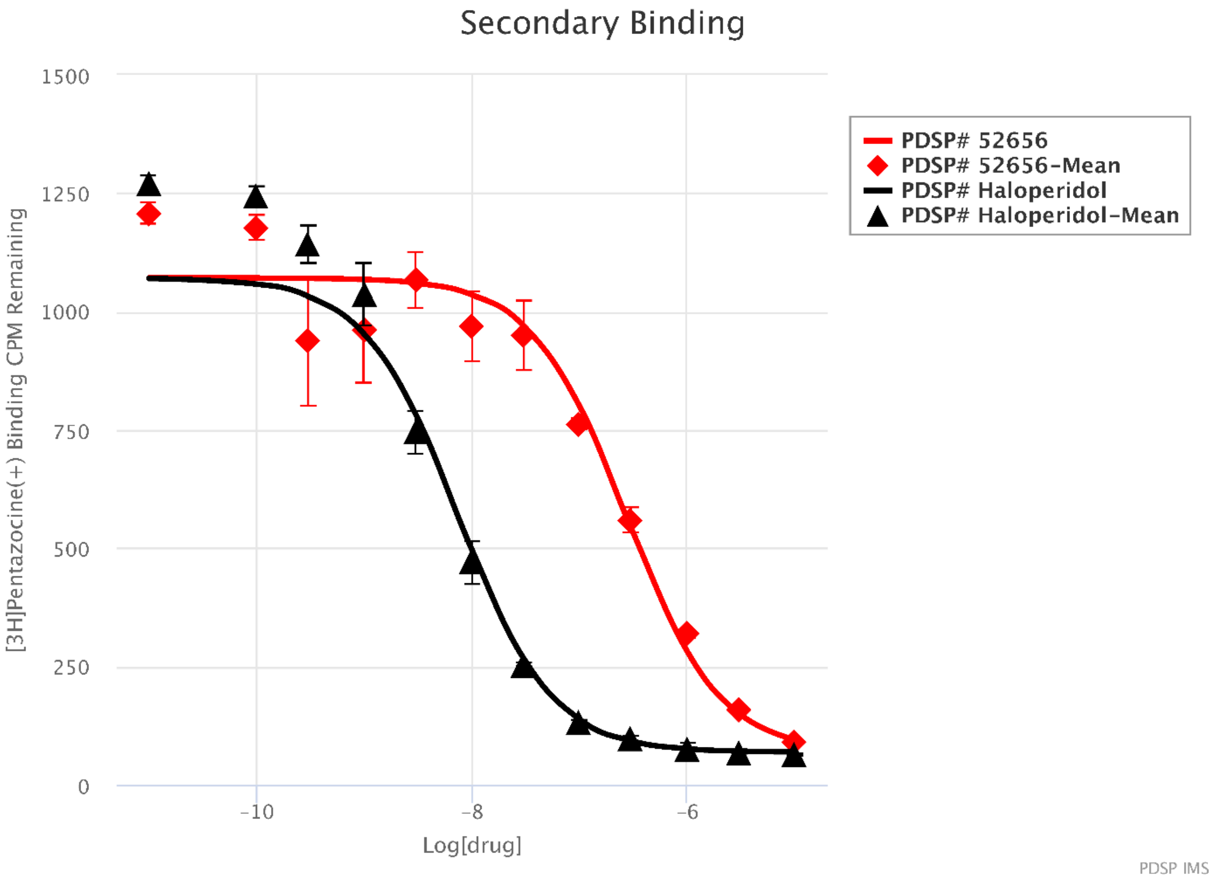

TMEM97 (Run 1)

PDSP #	LogKi	Ki (nM)
52656	-7.95	11.2
Haloperidol	-8.02	9.55

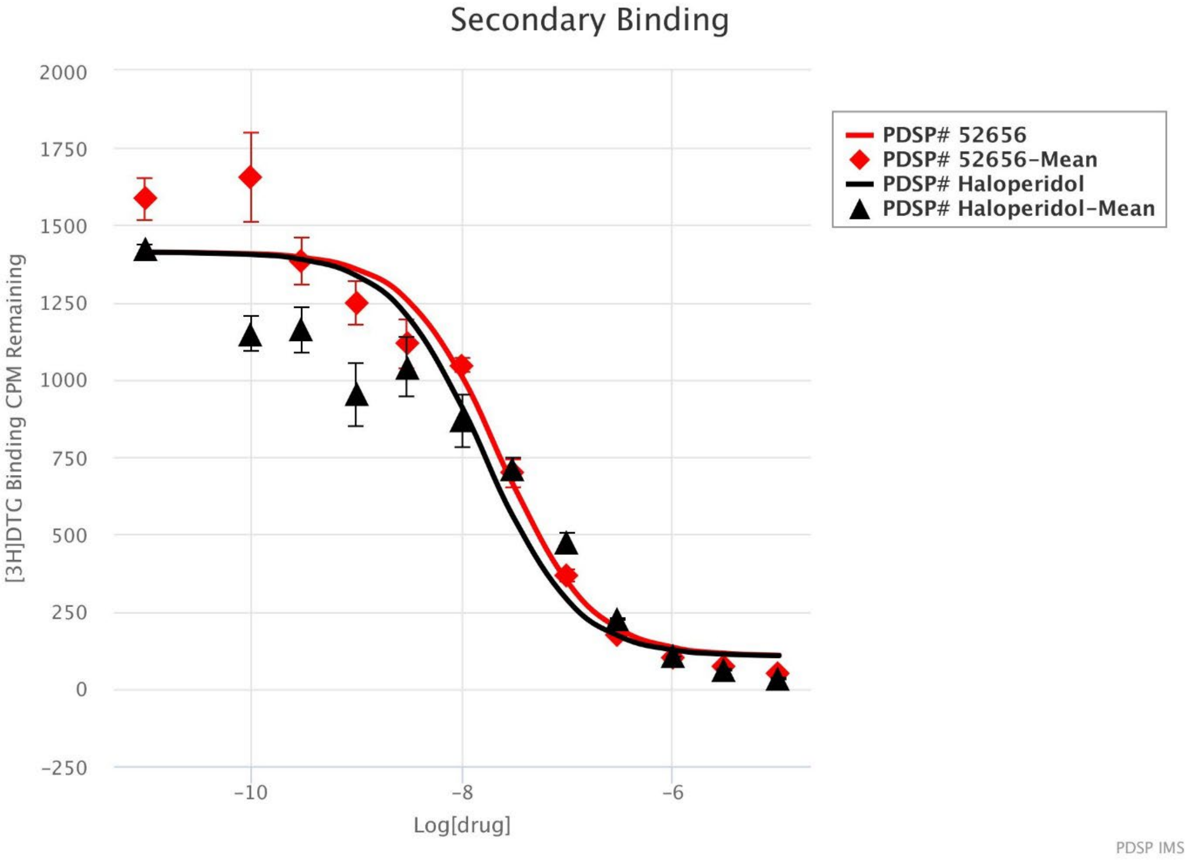

TMEM97 (Run 2)

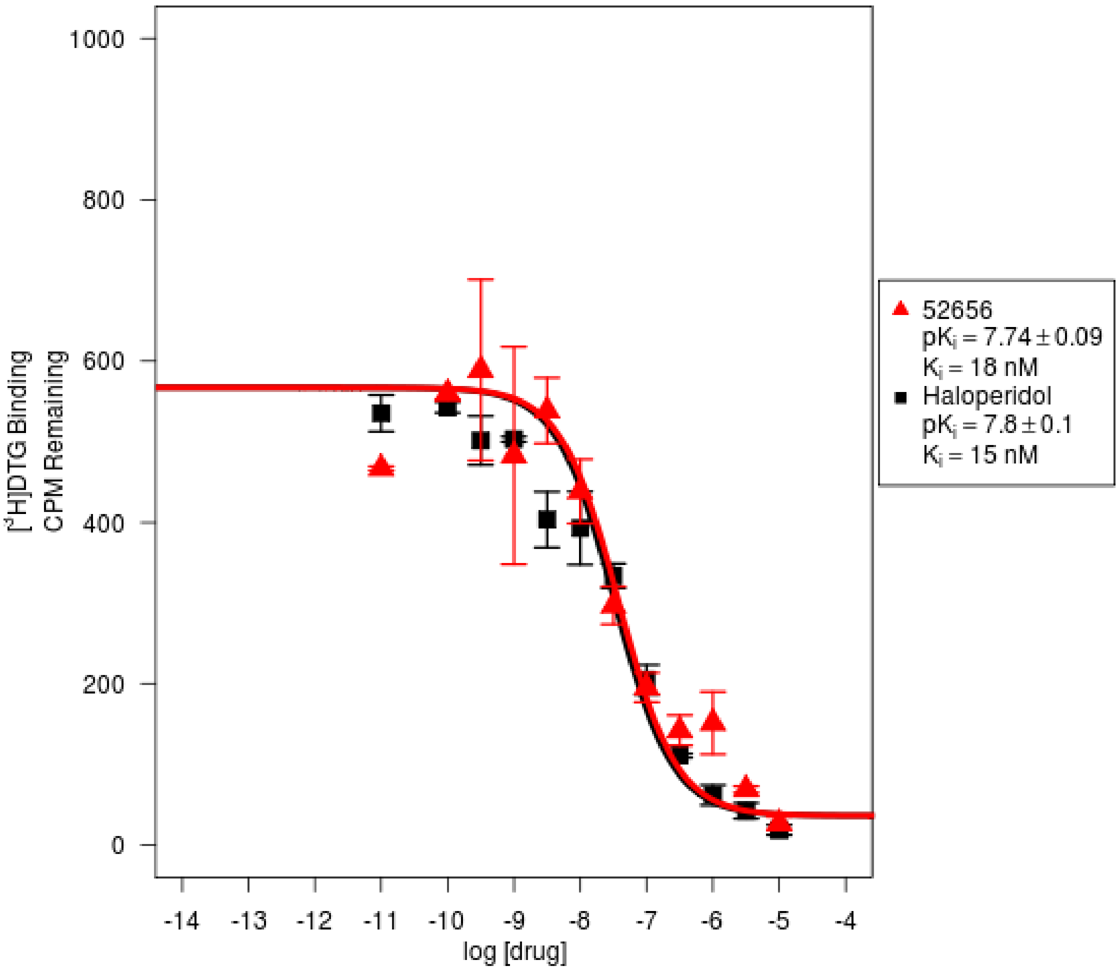

1000
800
600
400
200
0
[3H]DTG Binding
CPM Remaining
-14 -13 -12 -11 -10 -9 -8 -7 -6 -5 -4
log [drug]
52656
pK$_i$ = 7.74 ± 0.09
K$_i$ = 18 nM
Haloperidol
pK$_i$ = 7.8 ± 0.1
K$_i$ = 15 nM

TMEM97 (Run 3)

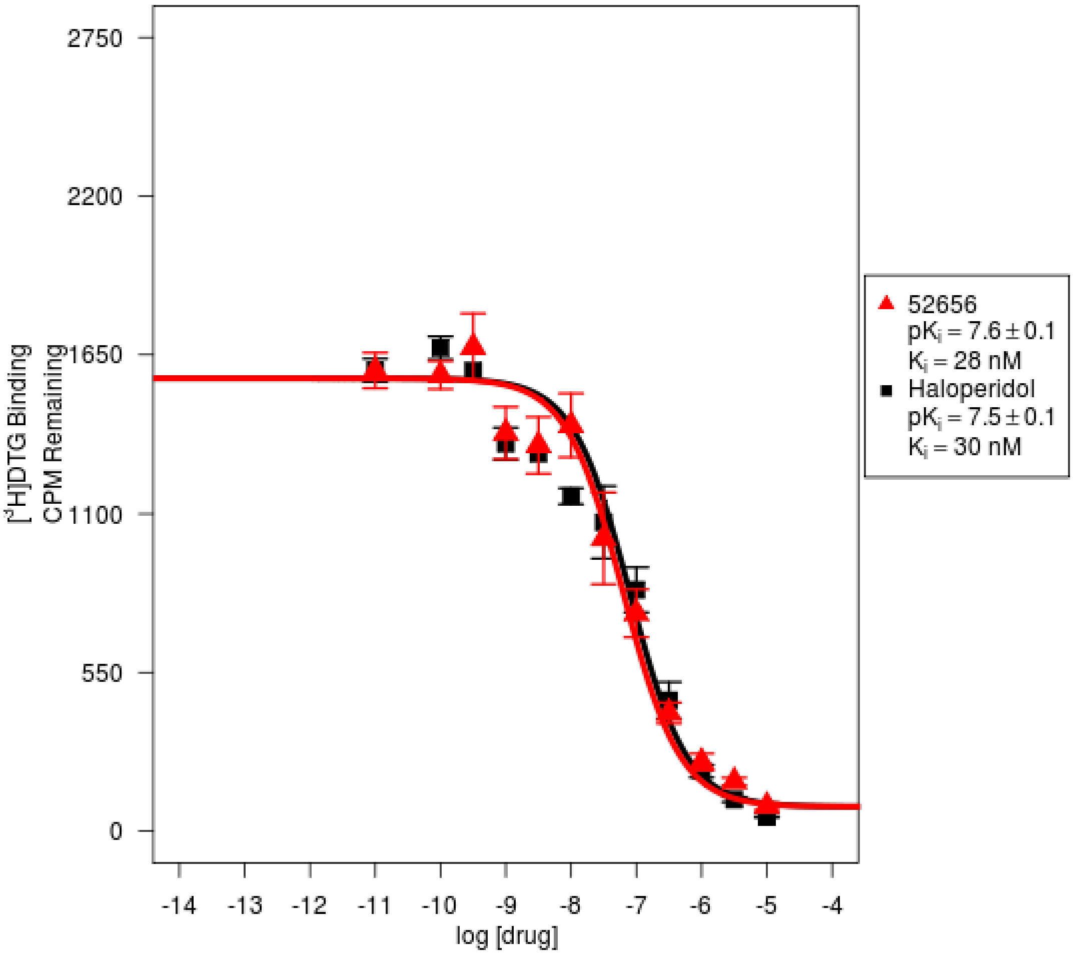
2750
2200
1650
1100
550
0
[3H]DTG Binding
CPM Remaining
-14
-13
-12
-11
-10
-9
-8
-7
-6
-5
-4
log [drug]
52656
pKi = 7.6 ± 0.1
Ki = 28 nM
Haloperidol
pKi = 7.5 ± 0.1
Ki = 30 nM

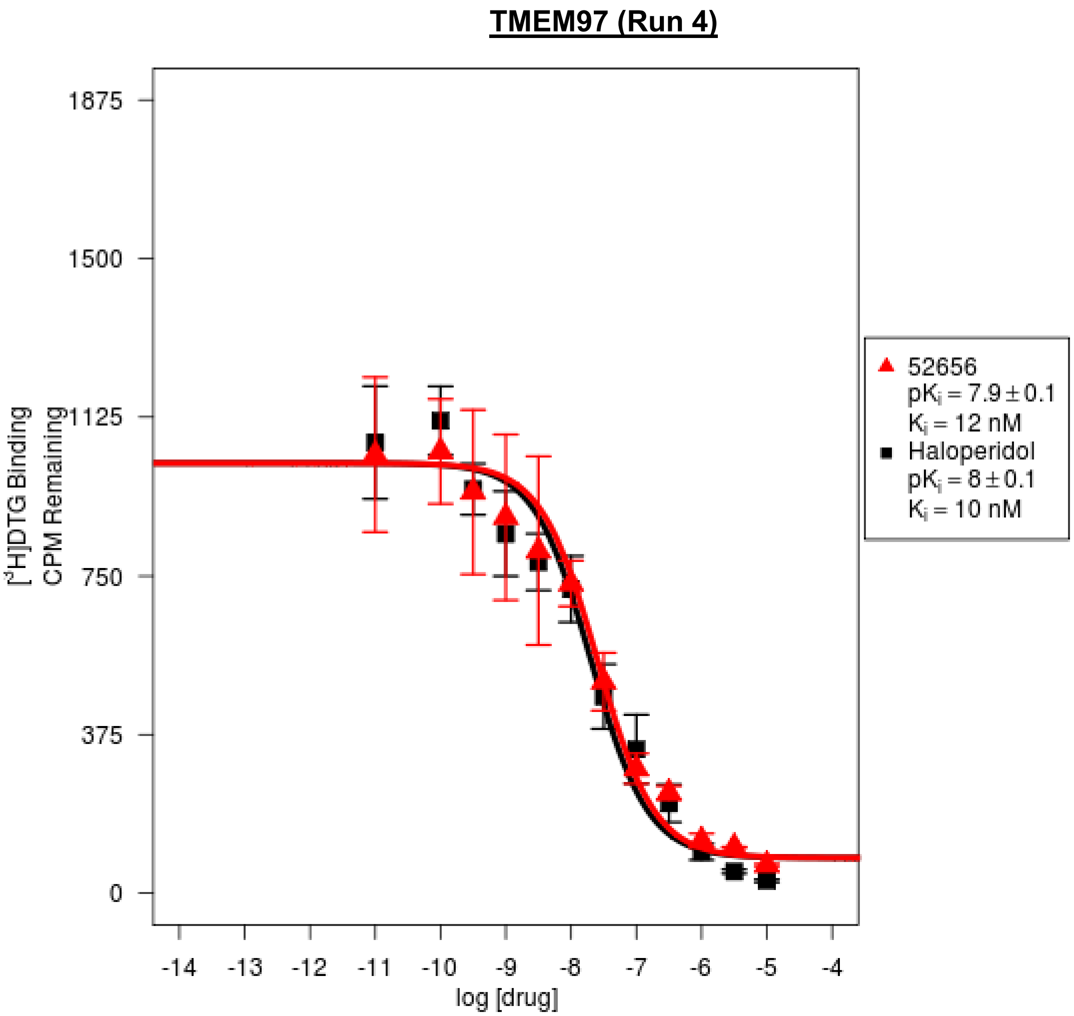
TMEM97 (Run 4)
1875
1500
1125
750
375
0
[3H]DTG Binding
CPM Remaining
-14
-13
-12
-11
-10
-9
-8
-7
-6
-5
-4
log [drug]
52656
pKi = 7.9 ± 0.1
Ki = 12 nM
Haloperidol
pKi = 8 ± 0.1
Ki = 10 nM

Alpha1A

PDSP #	LogKi	Ki (nM)
52656	-5.70	1994.34
Prazosin HCl	-9.57	0.27

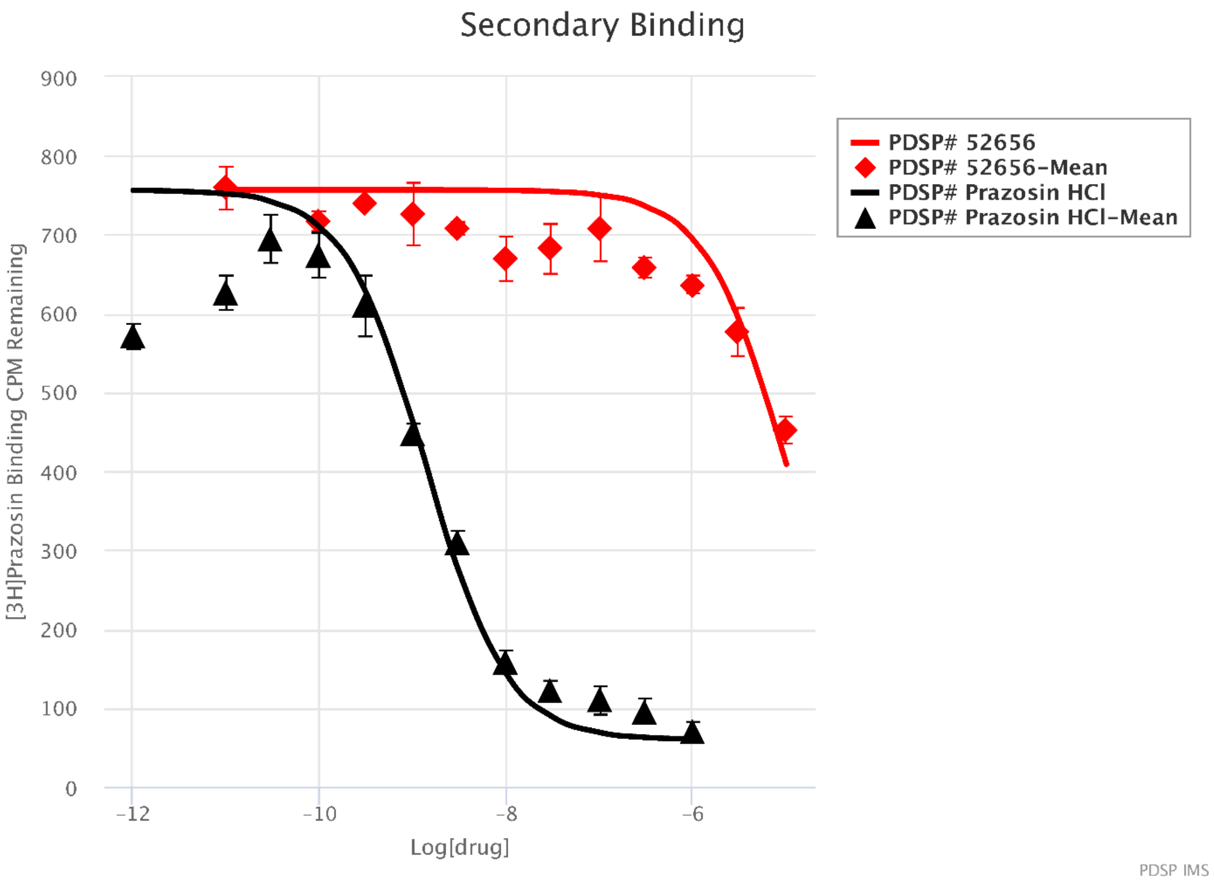

BZP Rat Brain

PDSP #	LogKi	Ki (nM)
52656	-5.18	6594.78
clonazepam	-8.89	1.29

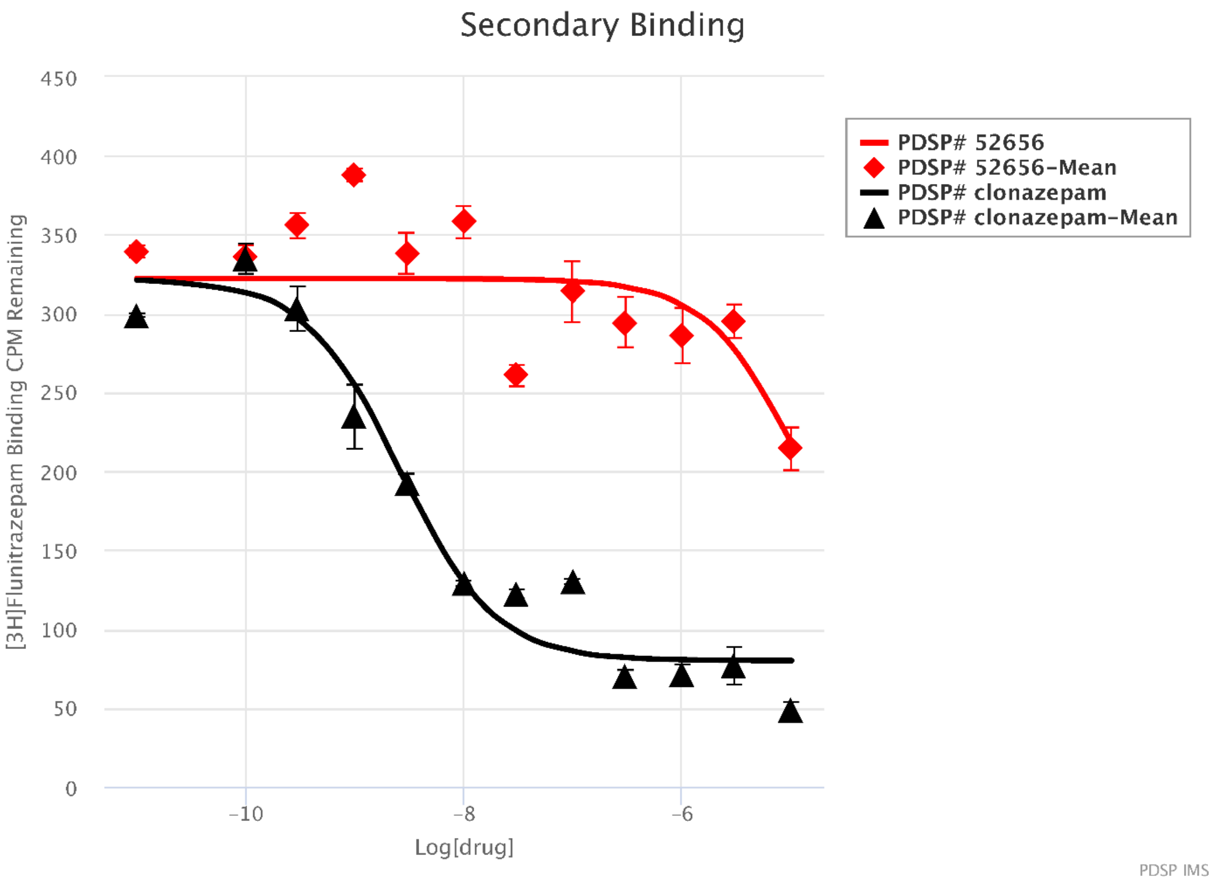

H4

PDSP #	LogKi	Ki (nM)
52656	-5.26	5511.88
Clozapine	-7.90	12.61

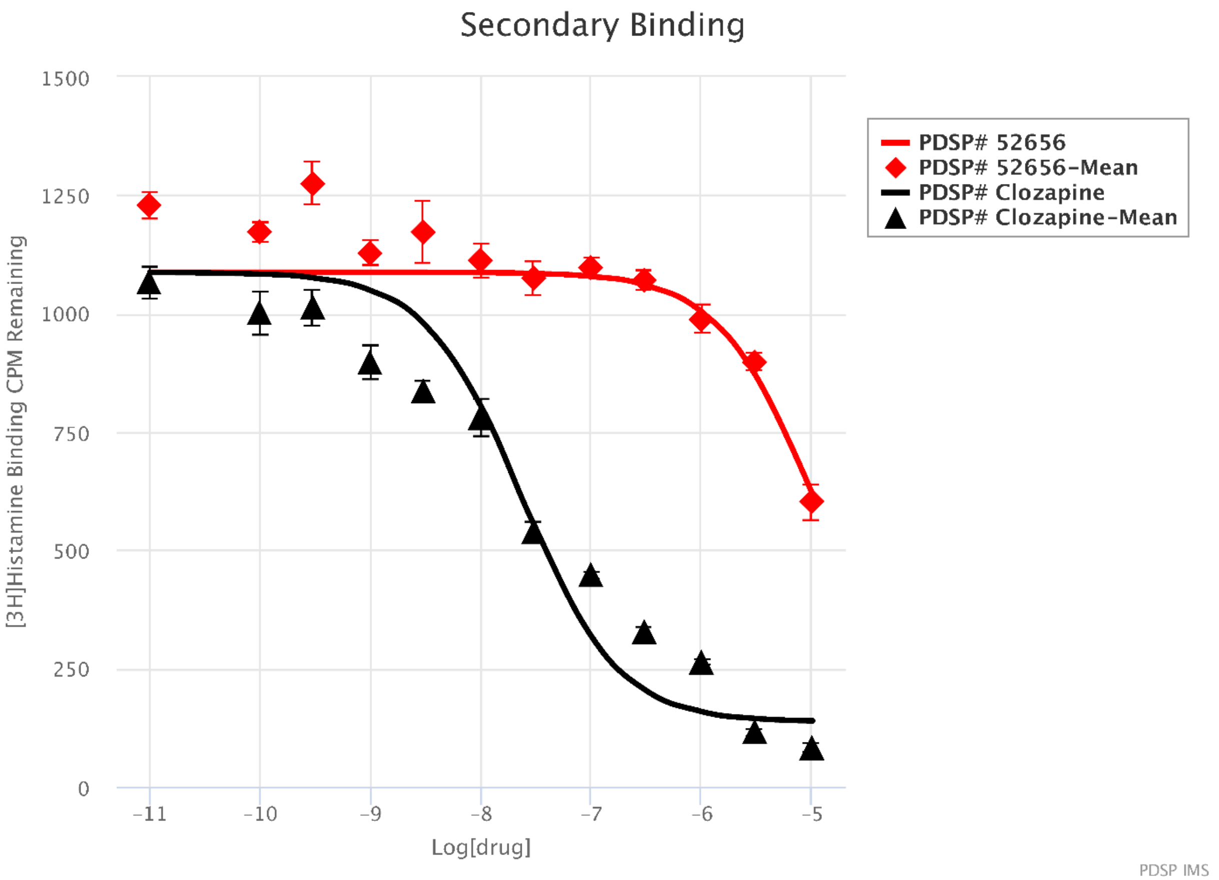

HERG

PDSP #	LogKi	Ki (nM)
52656	-6.08	839.07
Dofetilide	-7.61	24.31

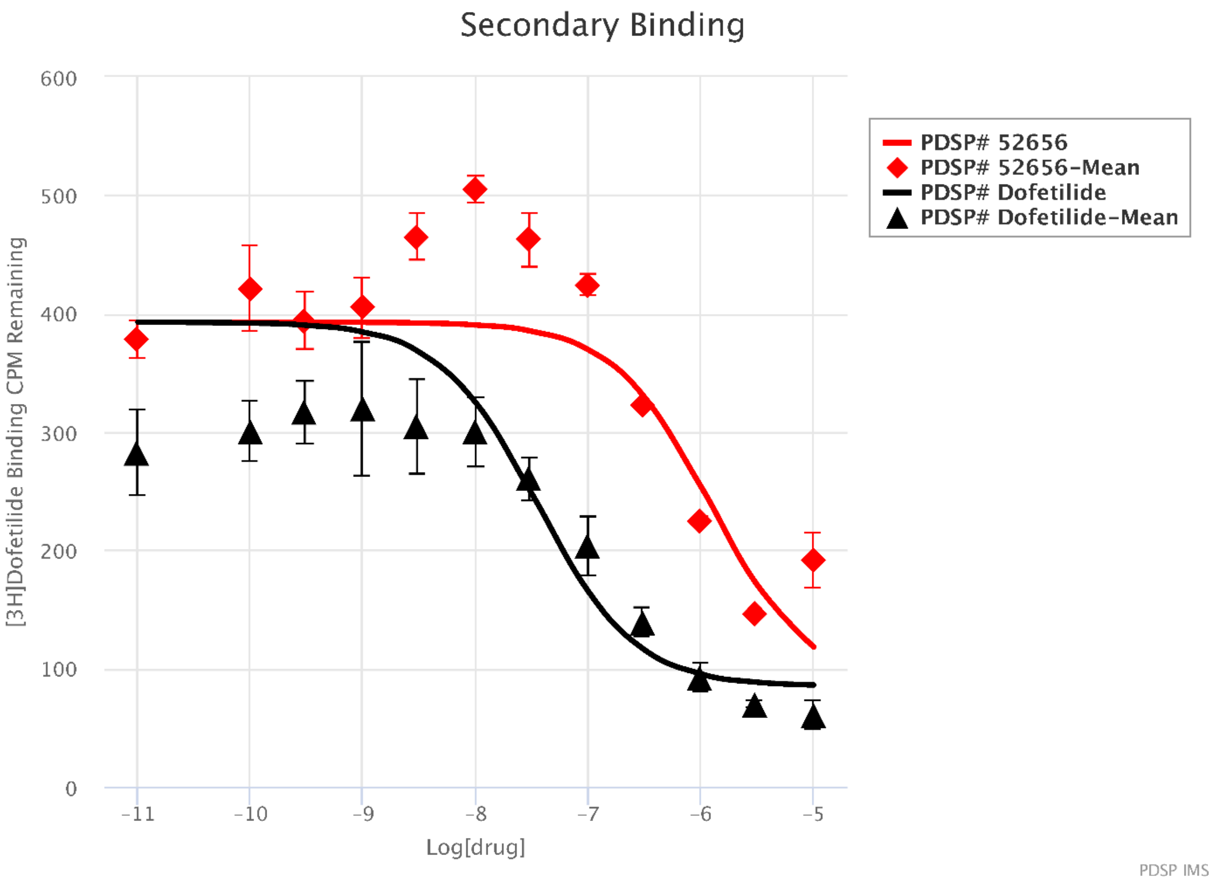

KOR (Run 1)

PDSP #	LogKi	Ki (nM)
52656	-5.33	4695.70
Salvinorin A	-8.33	4.71

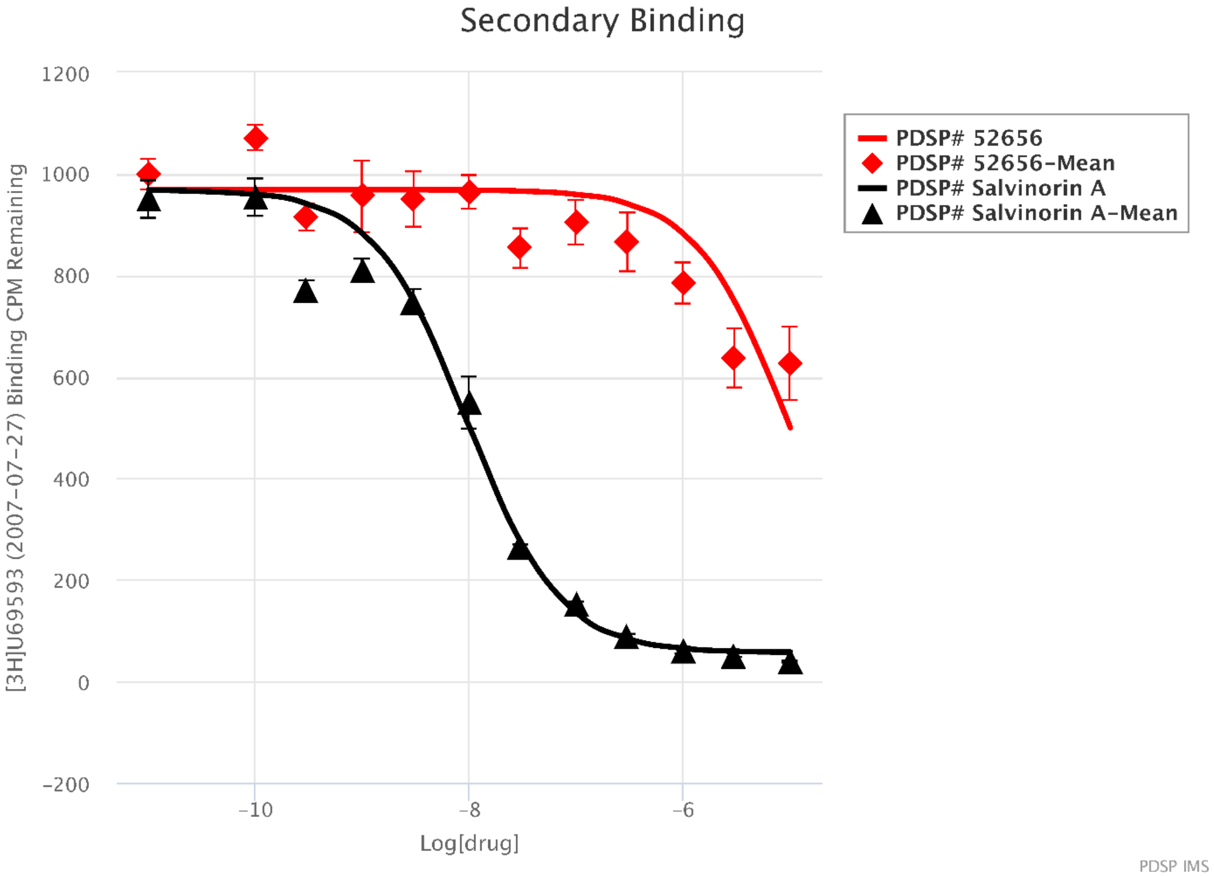

KOR (Run 2)

PDSP #	LogKi	Ki (nM)
52656	-5.02	9486.37
Salvinorin A	-8.14	7.19

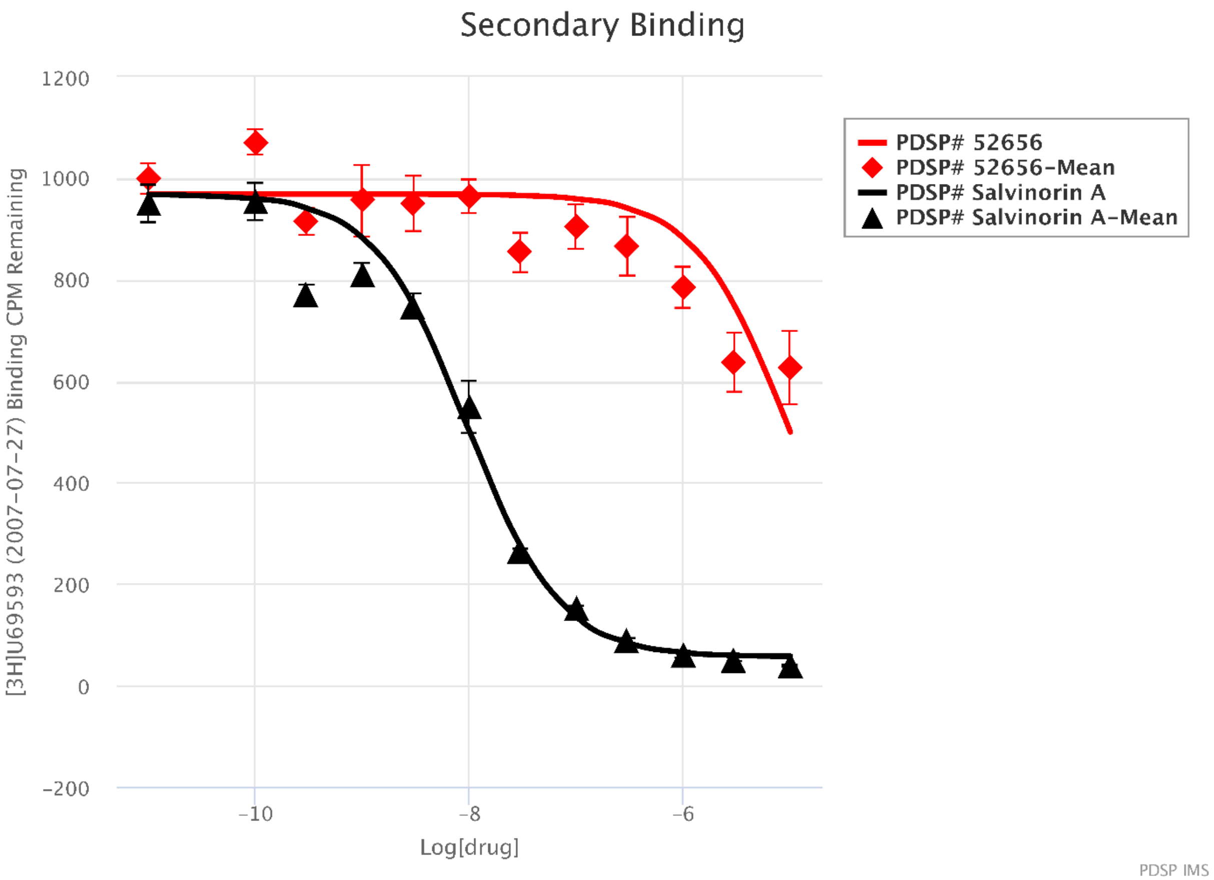

NET (Run 1)

PDSP #	LogKi	Ki (nM)
52656	-6.18	667.73
Desipramine HCl	-8.85	1.41

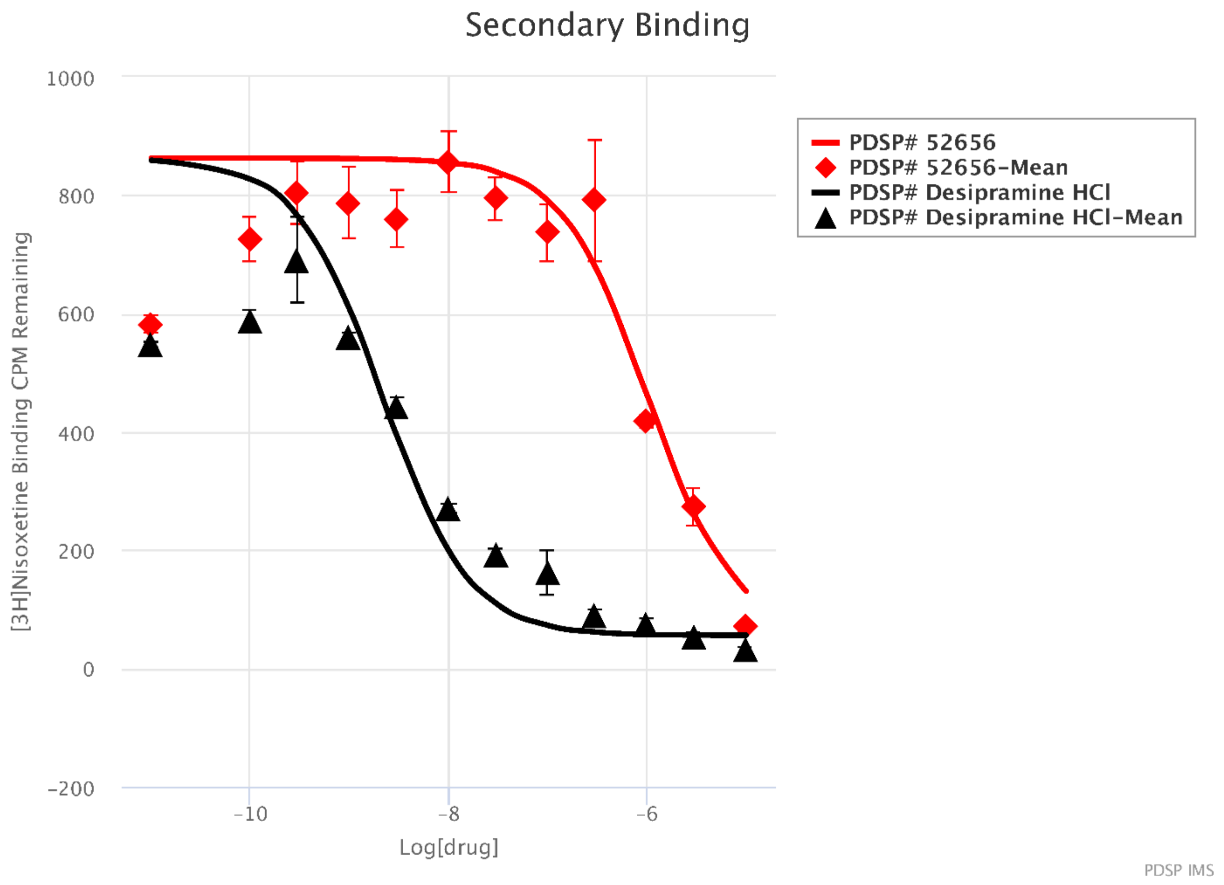

NET (Run 2)

PDSP #	LogKi	Ki (nM)
52656	-6.65	221.46
Desipramine HCl	-8.93	1.17

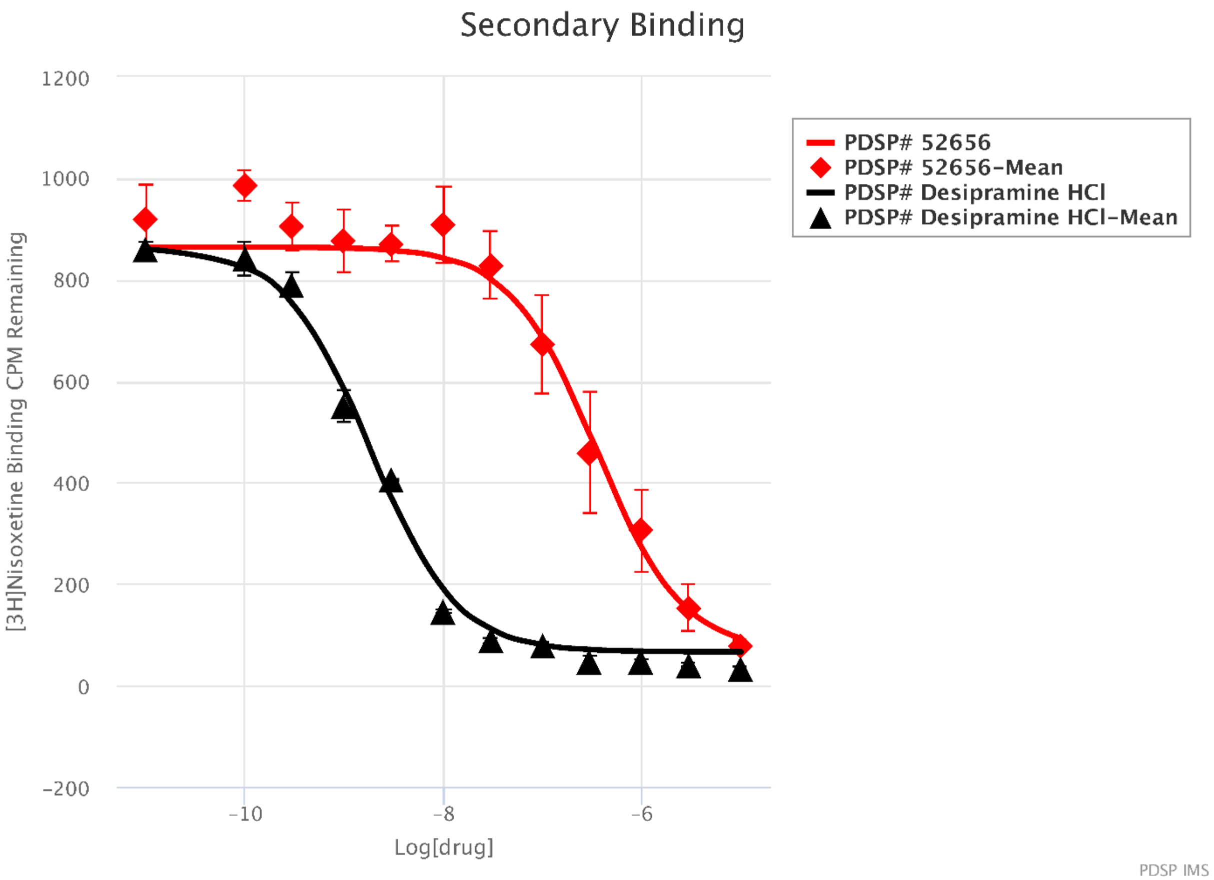

NET (Run 3)

PDSP #	LogKi	Ki (nM)
52656	-6.68	209.36
Desipramine HCl	-8.77	1.70

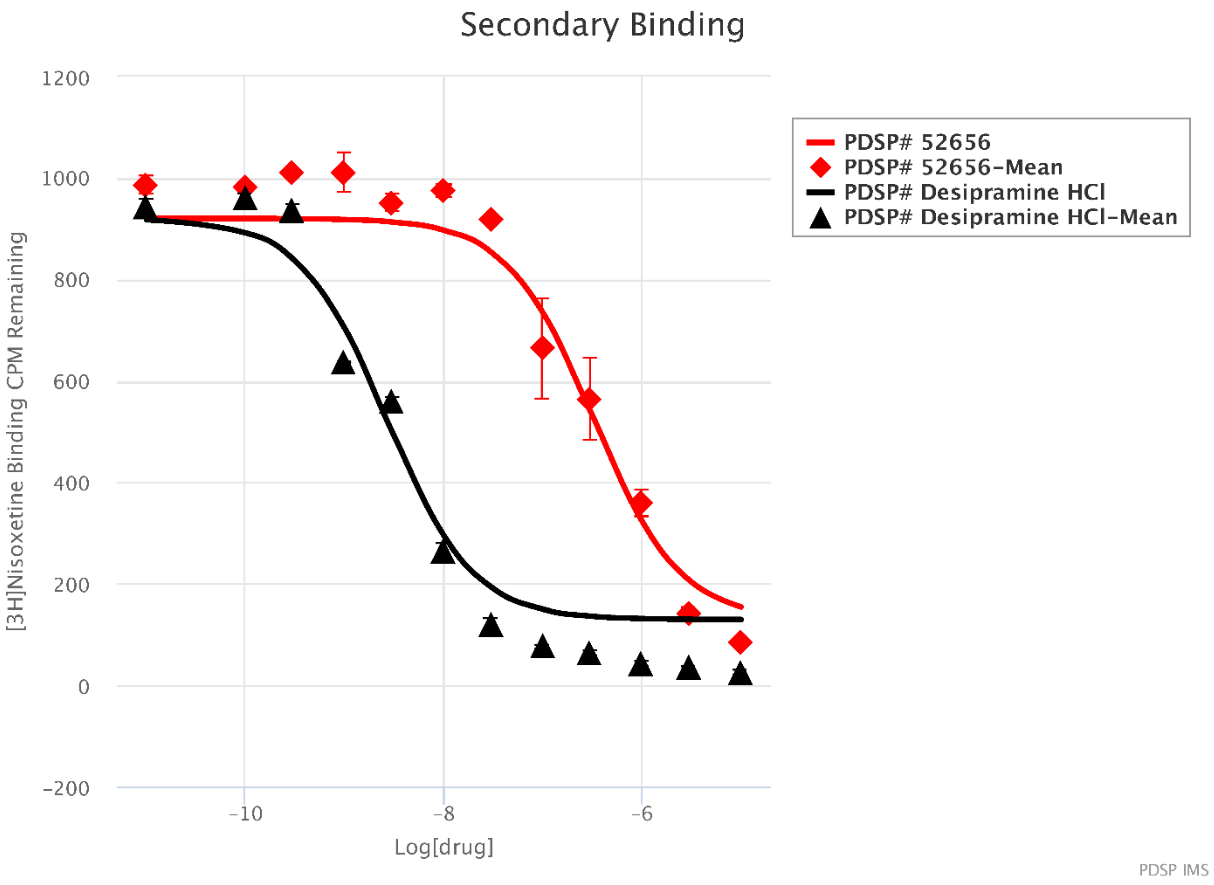

NET (Run 4)

PDSP #	LogKi	Ki (nM)
52656	-6.29	518.68
Desipramine HCl	-9.03	0.93

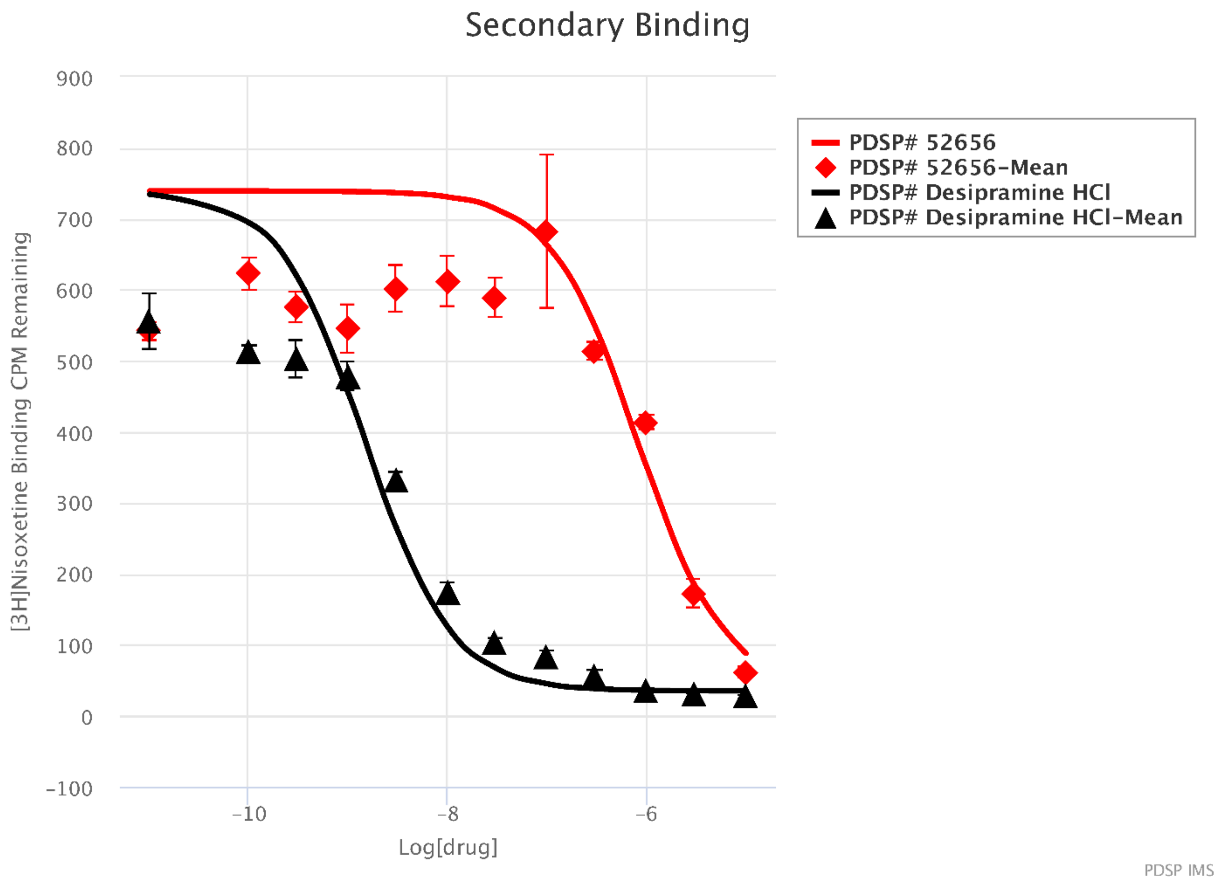

NMR Data for FEM-1689

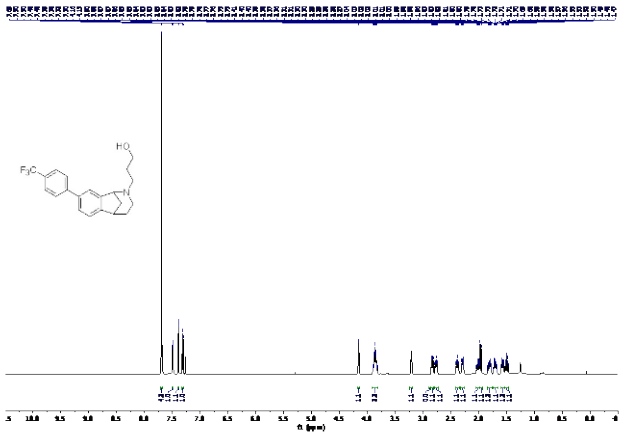

^{1}H NMR spectrum in $CDCl_3$

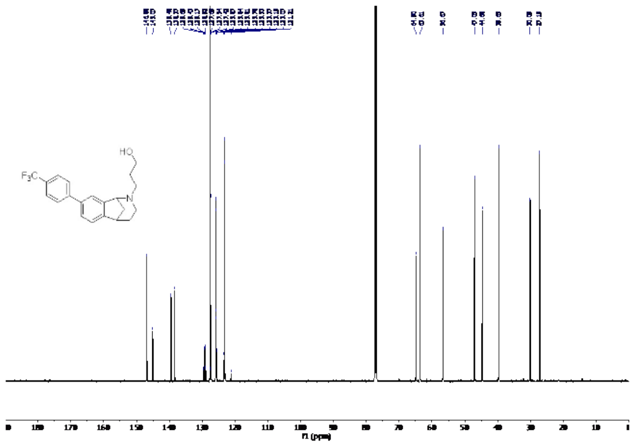

^{13}C NMR spectrum in $CDCl_3$

LCMS data for FEM-1689

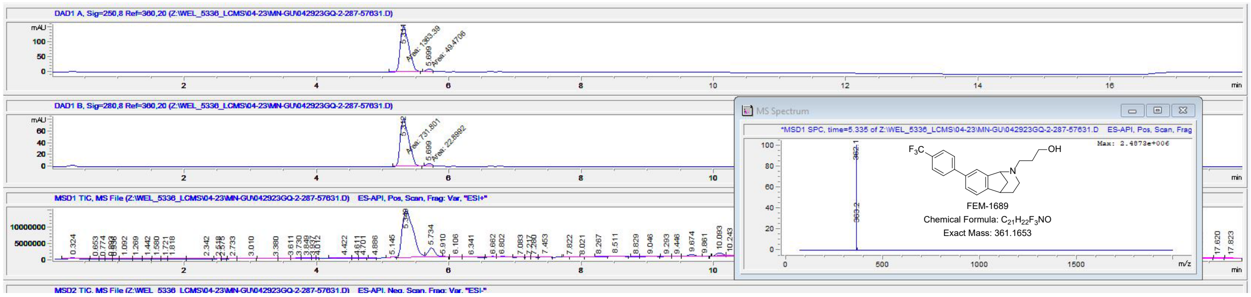

Retention Time, 5.31 min, purity 96.5

References

1. A. Zeisel *et al.*, Molecular Architecture of the Mouse Nervous System. *Cell* **174**, 999-1014 e1022 (2018).
2. S. A. Bhuiyan *et al.*, Harmonized cross-species cell atlases of trigeminal and dorsal root ganglia. *bioRxiv* 10.1101/2023.07.04.547740 (2023).
3. J. Besnard *et al.*, Automated design of ligands to polypharmacological profiles. *Nature* **492**, 215-220 (2012).
4. A. Alon *et al.*, Structures of the sigma2 receptor enable docking for bioactive ligand discovery. *Nature* **600**, 759-764 (2021).
5. H. Wang *et al.*, σ2R/TMEM97 in retinal ganglion cell degeneration. *Scientific Reports* **12** (2022).
6. R. P. Bonin, C. Bories, Y. De Koninck, A Simplified Up-Down Method (SUDO) for Measuring Mechanical Nociception in Rodents Using von Frey Filaments. *Molecular Pain* **10**, 1744-8069-1710-1726 (2014).
7. K. Hargreaves, R. Dubner, F. Brown, C. Flores, J. Joris, A new and sensitive method for measuring thermal nociception in cutaneous hyperalgesia. *Pain* **32**, 77-88 (1988).
8. M. V. Valtcheva *et al.*, Surgical extraction of human dorsal root ganglia from organ donors and preparation of primary sensory neuron cultures. *Nat Protoc* **11**, 1877-1888 (2016).
9. T. A. Ferreira *et al.*, Neuronal morphometry directly from bitmap images. *Nature Methods* **11**, 982-984 (2014).
10. M. S. Yousuf *et al.*, Endoplasmic reticulum stress in the dorsal root ganglia regulates large-conductance potassium channels and contributes to pain in a model of multiple sclerosis. *FASEB J* **34**, 12577-12598 (2020).
11. S. Megat *et al.*, Nociceptor Translational Profiling Reveals the Ragulator-Rag GTPase Complex as a Critical Generator of Neuropathic Pain. *J Neurosci* **39**, 393-411 (2019).
12. J. J. Sahn, S. F. Martin, Expedient synthesis of norbenzomorphan library via multicomponent assembly process coupled with ring-closing reactions. *ACS Comb Sci* **14**, 496-502 (2012).
13. T. A. Halgren *et al.*, Glide: a new approach for rapid, accurate docking and scoring. 2. Enrichment factors in database screening. *J Med Chem* **47**, 1750-1759 (2004).
14. Y. Lu, Q. Gu, S. F. Martin, Structure-affinity relationships of stereoisomers of norbenzomorphan-derived σ(2)R/TMEM97 modulators. *Eur J Med Chem* **257**, 115488 (2023).
